# Screening and Comparative Genomics of Probiotic Lactic Acid Bacteria from Bee Bread of *Apis Cerana*: Influence of Stevia and Stevioside on Bacterial Cell Growth and the Potential of Fermented Stevia as an Antidiabetic, Antioxidant, and Antifungal Agent

**DOI:** 10.3390/microorganisms13020216

**Published:** 2025-01-21

**Authors:** Samra Basharat, Lixin Zhai, Fuyao Jiang, Tanzila Asjad, Adil Khan, Xiangru Liao

**Affiliations:** 1 Key Laboratory of Industrial Biotechnology, Ministry of Education, Jiangnan University, Wuxi 214122, China; skysseven250@gmail.com (S.B.); 7180201041@stu.jiangnan.edu.cn (F.J.); tanzilaasjad483@gmail.com (T.A.); khanmalikadil@gmail.com (A.K.); 2 Henan Key Laboratory of Biomarker Based Rapid-Detection Technology for Food Safety, Institute of Molecular Detection Technology and Equipment, Xuchang University, Xuchang 461000, China; zhailixin216831@163.com

**Keywords:** bee bread, lactic acid bacteria, probiotic ability, stevia, steviosides, fermentation, fungal inhibition

## Abstract

The purpose of this research is to identify and characterize lactic acid bacteria (LAB) species in bee bread produced by honey bees (*Apis Cerana*) in the east mountain area of Suzhou, China. We isolated three strains, *Apilactobacillus kunkeei* (S1), *Lactiplantibacillus plantarum* (S2), and *Lacticaseibacillus pentosus* (S3), with S2 producing the highest amount of lactic acid. Phylogenetic analysis indicated that these isolates, along with the type strain, formed a distinct sub-cluster within the LAB group. The strains exhibited non-hemolytic activity, lacked functional virulence factors, demonstrated high acid and bile tolerance, strong adhesion to intestinal cells, and antimicrobial activity against pathogens, collectively indicating their safety and high probiotic potential for therapeutic applications. Our studies demonstrated that S2 and S3 grew well in the presence of stevia leaf powder and steviosides, while S1 showed reduced growth and inhibitory effects. Importantly, the stevia-fermented strains exhibited strong probiotic potential along with significant antidiabetic, antioxidant, and antifungal properties in vitro. These findings highlight their potential applications in the food, feed, and pharmaceutical industries. Future research should focus on in vivo experiments to validate these results and evaluate compatibility among the strains before their application in functional foods.

## 1. Introduction

The microbiome of honey bees plays a pivotal role in supporting various physiological and biochemical processes essential for the health of the colony. It is particularly important in the metabolism of food resources such as pollen and nectar, which provide essential nutrients [1]. Bee bread, a unique product formed through the lactic acid fermentation of pollen by bees in the hive, has been recognized as a significant source of nutrition. It contains a rich array of vitamins, proteins, polyphenols (including flavonoids [2], flavonols, and phenolic acids), and other bioactive compounds that contribute significantly to its nutritional value [3].

Bees are associated with various lactic acid bacteria (LAB) species, including LAB, *Enterococcus*, and *Bifidobacterium*, which are widely utilized in the food industry for their ability to produce lactic acid. The growth rate and biomass yield of LAB are influenced by factors like pH, temperature, medium composition, oxygen levels, and carbon sources [4,5]. Bee bread also hosts a diverse microbiota, including LAB, *Bacillus, yeasts*, and *molds*, which aid in its fermentation and preservation [6,7]. Among these, LAB species such as *Apilactobacillus kunkeei, Lactiplantibacillus plantarum*, and *Lacticaseibacillus pentosus* are particularly important for their roles in fermentation and potential health-promoting effects [8]. Despite growing interest, the biochemical mechanisms driving bee bread maturation, especially the role of LAB in fermentation, are not yet fully understood [9,10]. Recent studies have isolated several LAB strains from bee bread to better understand their functional properties. These LAB strains are known for their probiotic properties, including their ability to enhance digestion, improve nutrient absorption, and provide protection against harmful bacteria [11,12,13]. Additionally, LAB are increasingly recognized for their potential use in food preservation, particularly due to their ability to produce antimicrobial substances such as lactic acid and bacteriocins, which inhibit the growth of spoilage microbes and mycotoxins [14,15].

Stevia extracts have been utilized by traditional South American cultures for many years due to their health benefits. Stevia glycosides and other phytochemicals, including phenolic compounds, have demonstrated significant biological activity, indicating their potential therapeutic value [16,17,18]. *Stevia rebaudiana*, the source of these sweeteners, contains steviosides, compounds that are 200–300 times sweeter than sucrose [19,20]. Stevia is not only valued for its sweetening properties, but also its potential therapeutic benefits [19,21], including its ability to manage blood sugar levels, improve hypertension, and exhibit antibacterial and anti-tumor effects [22,23,24]. Despite these benefits, the effects of steviosides and other Stevia compounds on the growth and metabolic activity of probiotic LAB remain underexplored, particularly in terms of their impact on lactic acid production and fermentation processes [25]. Interestingly, the connection between Stevia and lactic acid bacteria adds another dimension to the dynamics of bee bread fermentation. Stevia, particularly *Stevia rebaudiana*, produces natural sweeteners like steviosides [26]. These steviosides have become increasingly popular in functional foods due to their low-calorie content and potential health benefits [27]. However, the interaction between stevia compounds and LAB remains an underexplored area of research, particularly in the context of bee bread [28].

The aim of this study was to investigate the effects of stevia leaf powder and steviosides on the growth and lactic acid production of three LAB strains—*Apl. kunkeei* (S1), *Lpb. plantarum* (S2), and *Lcb. pentosus* (S3)—which were isolated from bee bread collected from Suzhou, China. The strains *Apl. kunkeei*, *Lpb. plantarum*, and *Lcb. pentosus* were specifically selected for this study due to the lack of prior research on the effects of Stevia-derived compounds, particularly steviosides, on these particular strains. While Stevia has been widely studied for its impact on various probiotics, its influence on these specific LAB strains, which are commonly found in bee bread and play crucial roles in fermentation and food preservation, remains unexplored. This gap in the literature presents an opportunity to investigate the potential metabolic, probiotic, and fermentation-enhancing effects of Stevia on these LAB strains.

Moreover, this research seeks to examine how Stevia-derived compounds influence the metabolism of these LAB strains, specifically focusing on their ability to produce lactic acid, a key metabolite in fermentation. We hypothesize that steviosides may stimulate the growth and fermentation activity of these LAB strains, enhancing the quality and nutritional value of bee bread and other fermented products. Additionally, this study explores the potential of these LAB strains to degrade mycotoxins and inhibit fungal growth, a critical concern in food and feed safety. Mycotoxin contamination, primarily from fungal species in the genera Aspergillus, Penicillium, and Fusarium, poses significant risks to human and animal health, including carcinogenic, mutagenic, and immunotoxic effects. LAB have been shown to possess mycotoxin-degrading capabilities, and their production of antimicrobial metabolites can inhibit fungal growth, thereby contributing to food preservation and safety [25,29].

This study employs modern molecular techniques, including whole genome sequencing (WGS) and 16S rDNA sequencing, to analyze the genetic diversity of the LAB strains and explore their functional properties. By investigating the genomic basis for lactic acid production and antifungal activity, this research aims to provide insights into the potential applications of these LAB strains in the food and agricultural industries. Furthermore, the findings could offer a theoretical foundation for the industrial production of artificial bee bread and other fermented products, enhancing their safety, nutritional value, and health benefits. Overall, this research seeks to elucidate the complex interactions between Stevia-derived compounds, honey bee (products) fermentation, and lactic acid bacteria in the fermentation process, with the broader goal of improving food safety, fermentation quality, and nutritional outcomes. By expanding our understanding of the biochemical pathways involved in LAB fermentation and the potential of stevia as a functional food ingredient, this study aims to contribute to the growing body of knowledge surrounding functional foods and probiotics.

## 2. Materials and Methods

### 2.1. Sampling of Bee Bread

Four types of bee bread samples were collected from four different habitats in the East Mountains of Suzhou, Jiangsu Province, China, in August 2022. The samples were obtained from a professional beekeeper and extracted using a 25 mL sterile syringe. After collection, each sample was stored in a 50 mL sterile glass-sealed container. The samples were transported at low temperatures and immediately analyzed at Jiangnan University’s lab in Wuxi, China. Samples A1–4 were refrigerated at 4 °C for temporary storage.

### 2.2. Isolation of Lactic Acid Bacteria from Bee Bread

From five to eight grams of bee bread (BB) samples were homogenized in a sterile 0.2% peptone-water solution using a BagMixer 400 (Interscience, Shanghai, China) for 5 min at room temperature. Serial dilutions were then plated on both MRS and FYP agar. The FYP agar composition included 10 g of D-fructose (Shanghai Aladdin Biochemical Technology Co., Ltd., Shanghai, China), 10 g of yeast extract, 5 g of polypeptone, 2 g of sodium acetate, and 0.5 g of Tween 80 (Shanghai Macklin Biochemical Co., Ltd., Shanghai, China). This agar solution was supplemented with 0.2 g of MgSO_4_·7H_2_O (Shanghai Chemical Reagent Co., Ltd., Shanghai, China), 0.01 g of FeSO_4_·7H_2_O, 0.01 g of MnSO_4_·4H_2_O, 0.01 g of NaCl (Shanghai Lingfeng Chemical Reagent Co., Ltd., Shanghai, China), 0.05 g of cycloheximide, and 0.05 g of sodium azide (Shanghai Aladdin Biochemical Technology Co., Ltd.) per liter of distilled water, adjusted to pH 6.8. The plates were incubated aerobically at 30 °C for 24 to 48 h with the addition of 0.5% CaCO_3_ (*w*/*v*) [30]. Acid-producing bacterial colonies were selected due to their surrounding transparent circles. MRS agar medium was inoculated with these colonies. The calcium-soluble circle was located where the culture medium transitioned from cloudy to clear. The strain led to the production of acid, resulting in the dissolution of CaCO_3_. The selection process was carried out a total of 3–4 times. The pure bacterial isolates were stored in nutrient broth containing 20% (*v*/*v*) glycerol at −80 °C [31].

### 2.3. Identification of Strains

MRS and FYP broth (Oxoid, Hampshire, UK) aided as enrichment media. From each low-dilution MRS and FYP plate, the 10–15 colonies with the best appearance and largest clearance zones were selected. The agar medium was inoculated with streaked colonies. A clear zone indicates the ability of certain bacteria to dissolve CaCO_3_ through the organic acids they produce. After incubating in FYP broth at 30 °C for 24 h, the Gram-positive catalase-negative isolates were streaked onto FYP agar.

### 2.4. Morphologic and Biochemical Characterization

Prior to each assay, LAB isolates were sub-cultured twice in MRSB. The morphology and biochemistry of the LAB colonies were analyzed based on Bergey’s Manual of Determinative Bacteriology (Carolina Biological Supply, Catalog No. S07431ND). All strains underwent biochemical assays, which included Gram-staining, starch hydrolysis, bile salt hydrolase activity, indole generation, catalase testing, H2S production, gelatin liquefaction, and carbohydrate fermentation (reagents for these assays were sourced from Shanghai Aladdin Biochemical Technology Co., Ltd.). This comprehensive approach ensures accurate identification and characterization of the LAB isolates used in our study [32]. Three different incubation temperatures (18, 37, and 45 °C) were tested to determine the ideal temperature for the strain’s growth [33,34].

### 2.5. Molecular Identification Using 16S rRNA Gene Sequencing

To identify the isolated bacterial strains, genomic DNA was extracted and stored in 150 microliters of double-distilled water at −24 °C. DNA purity was evaluated by measuring absorbance ratios at 260 and 280 nm using a spectrophotometer (Thermo Scientific, Shanghai, China). The 16S rRNA gene was amplified using the primers 24F (AGA GTT TGA TGG CT) and 1541R (AAG GAG GTG ATC CCG CA) in a 50 µL PCR mixture containing bacterial DNA, Taq DNA polymerase (Thermo Scientific), and de-ionized water (Shanghai Lingfeng Chemical Reagent Co., Ltd.). The PCR conditions included an initial denaturation step of 3 min, followed by 35 cycles of denaturation at 95 °C for 30 s, annealing at 50 °C for 30 s, and extension at 72 °C for 1.5 min (Table 1). A final extension was performed at 72 °C for 10 min. The PCR products were analyzed by electrophoresis on a 1% agarose gel containing 5.5 g/mL ethidium bromide (Shanghai Aladdin Biochemical Technology Co., Ltd.), visualized under a UV transilluminator.

The 16S rRNA gene sequences were aligned using the NCBI BLASTn tool (https://www.ncbi.nlm.nih.gov/) to identify the closest related genera. The strains showed the highest similarity to *Lcb. pentosus* (99.47%), *Lpb plantarum* (99.79%), and *Apb. kunkeei* (99.37%). Sequence editing was performed with BioEdit version 7.2.5, and a phylogenetic tree was constructed using the neighbor-joining method in MEGA 11.0.8 Software. The 16S rRNA gene sequences were deposited in GenBank, with accession numbers CP128865, CP134769, and CP134770 for *Apb. kunkeei* (S1), *Lactiplantibacillus plantarum* strain (S2), and *Lcb. pentosus* (S3), respectively. Since *Lpb. plantarum* and *Lcb. pentosus* exhibit a high identity (>99%) in their 16S rRNA gene sequences, further species-level differentiation is challenging using this marker alone. This limitation highlights the necessity of complementary methods such as whole-genome sequencing for more accurate taxonomic identification.

### 2.6. Whole Genome Sequencing, Assembly, and Quality Control

Whole-genome sequencing of the isolated strains was performed using both second-generation Illumina and third-generation Nanopore sequencing platforms. The bacterial genomes were sequenced to a depth of ≥100X to ensure high-quality coverage. The sequencing data were assembled using Illumina short-read data (Q30 > 85%) and Nanopore long-read data to construct a comprehensive genome map. The assembly yielded no gaps, except for minor exceptions noted in the contract. Plasmid sequences were not included in the final analysis. To improve the accuracy of the genome assembly, Illumina reads were aligned to the contigs using the BWA (0.7.17) software. Long reads from Nanopore sequencing were aligned using Minimap2 (2.17), and sequencing depth statistics were calculated. The average sequencing depth was determined using Samtools (1.10), with a sliding window of 2000 bp. Contigs were further refined using Pilon (1.23) to ensure the accuracy of the genome assembly.

Following DNA extraction, the strains were cultured in MRS broth and grown for 24 h. Cells were harvested and washed three times with PBS and were then subjected to centrifugation. DNA integrity and concentration were measured using the Quant-iT PicoGreen dsDNA Assay Kit (Thermo Fisher Scientific, Shanghai, China), and the DNA samples were stored at −20 °C for sequencing. For the genomic library preparation, the standard Illumina TruSeq Nano DNA LT protocol was followed. High-quality DNA libraries were prepared and analyzed using Nanodrop, Qubit, and 0.35% agarose gel electrophoresis to ensure intact genomic DNA. Illumina sequencing produced high-accuracy reads which were used for genome assembly and functional annotation. Finally, the third-generation single-molecule sequencing data were processed with the HGAP method and CANU assembler to obtain final genomic sequences. These sequencing efforts resulted in the complete genome sequences of *Apl. kunkeei, Lpb. plantarum*, and *Lcb. pentosus*, which were deposited in GenBank for public access. The strains were submitted to the NCBI GenBank database under the names *Apl. kunkeei S1, Lpb. plantarum SYBC-SM2*, and *Lcb pentosus SYBC-SM3.* For simplicity in the manuscript, these strains are referred to as S1, S2, and S3, respectively.

#### 2.6.1. Gene Prediction Functional Annotation of the Genome

The Prokka software (v1.14.6) was used to predict coding genes from the assembled genome. This software is a collection of gene element prediction tools, including Prodigal (v2.6) for coding gene prediction, Aragorn (v1.2) for tRNA prediction, RNAmmer (1.2) for rRNA prediction, and Infernal (1.1) for miscellaneous RNA prediction. The predicted gene elements were then compiled and provisionally annotated. Function annotation was performed using various general databases, including Pfam, Refseq, Uniprot, Nr, Tigrfams, COG, KEGG, GO, and eight others. Additionally, proprietary databases such as ARDB, CAZy, CARD, CYP450, VFDB, TCDB, and signal peptide prediction were utilized. GO, KEGG, PFAM, NR, Swiss-Prot, and TreMBL were also employed. GO, KEGG, and PFAM were annotated using emapper 2.1.6, which relies on eggNOG, a comprehensive database, and diamond for database matching. Personalized analyses were conducted, including comparative genome analysis, comparison of sequenced samples with reference genomes, gene family construction, covariance analysis, shared/endemic gene analysis, species evolution analysis, and selection pressure analysis.

#### 2.6.2. Genotype Analysis of Carbohydrate Metabolism

The CAZy database was employed to identify carbohydrate-active enzymes in the annotated LAB genomes using HMMER-3.1. The clustering of LAB strains was carried out using HEMI version 1.0 software. The CAZy database focuses on the analysis of biochemical, genomic, and structural data related to carbohydrate enzymes [35,36]. Protein sequences were annotated based on the CAZy database using three tools, HMMER, Hotpep, and DIAMOND, respectively.

#### 2.6.3. Secondary Metabolite Gene Cluster Analysis

Secondary metabolites are substances synthesized by microorganisms during a certain growth period using primary metabolites as precursors that have no clear function in the life activities of the microorganism and are not essential for growth and reproduction. The genome was predicted using the anti-SMASH program (version 6).

#### 2.6.4. Repetitive Sequence and Pathogen-Host Interaction Analysis

PHI, which stands for Pathogen Host Interactions, is a database that focuses on pathogen–host interactions. The database includes experimentally validated information collected from fungal, oomycete, and bacterial pathogens infecting a wide range of hosts, such as animals, plants, fungi, and insects. In terms of prokaryotic genomes, the presence of repetitive sequences is quite minimal. To predict repeat sequences in bacterial genomes, we used the RepeatMasker (4.1.0) software.

#### 2.6.5. Genotype Analysis of Antibiotic Resistance

Information about resistance genes in each genome was extracted from the CARD database. The CARD database is structured as the Antibiotic Resistance Ontology (ARO), integrating information about antibiotic components, their targets, mechanisms of resistance, and gene variants. The CARD database was used to annotate the target proteins through Diamond blastp (v2.1.10).

#### 2.6.6. Cytochrome P450 and VFDB

CYPED was used to identify the large family of protein known as Cytochromes P450 (CYP450), whose cofactor is heme. The Virulence Factors Database (VFDB) provided details on virulence factors of bacterial pathogens. The protein sequences were annotated using Diamond blastp against VFDB.

#### 2.6.7. TCDB Transporter Proteins

The TCDB (github.com/SaierLaboratory) is a database for classifying membrane transport proteins. The target protein sequences were annotated using Diamond blastp based on the TCDB database.

#### 2.6.8. Identification of CRISPR-Cas Systems and Prophages

Using default settings, the CRISPR-CasFinder (4.3.2) tool was employed to detect CRISPR regions and associated (Cas) proteins [23]. PhiSpy (4.2.21) was used to identify and annotate the prophages in all strains [37,38,39].

### 2.7. Evaluation of In Vitro Probiotic Ability of Isolated Strains

#### 2.7.1. Acid and Bile Salts Tolerance

Oxgall was added to the MRS broth to prepare the bile solution. During the exponential growth phase, samples were taken and introduced into either artificial gastric juice or bile solution, then incubated at 37 °C for 2 h. After incubation, cultures were harvested and centrifuged to remove the supernatant. The resulting pellet was resuspended in PBS buffer (pH 7.2) and diluted with sterile distilled water to the desired concentration. The diluted mixture was plated on MRS agar and incubated at 37 °C for 24 h. The log CFU/mL count for individual colonies was then determined to assess viability [40].

Survival rate % = [Biomass at time (t)/Biomass at initial time (0)] ×100

#### 2.7.2. Simulated Human GI Tract Conditions

To prepare phosphate-buffered saline (PBS), 0.8% NaCl, 0.02% KH_2_PO_4_, and 0.115% Na_2_HPO_4_ (*w*/*v*) were mixed. The pH of the PBS was adjusted to 2.5, 3.0, and 4.0 using 1N HCl, followed by sterilization by autoclaving at 121 °C for 15–20 min. For simulated gastric juice, pepsin was added to the PBS buffer and filter-sterilized using a 0.25 mm filter membrane, achieving a final concentration of 5 g/L. To produce simulated intestinal juice, trypsin (1:250, Sinopharm Chemical Reagent Co., Ltd., Shanghai, China) was mixed with the PBS buffer solution, and the pH was adjusted to 7.0 using 1N NaOH before autoclaving at 121 °C for 15 min. Finally, a 1 g/L trypsin solution was added to the sterilized PBS buffer (pH 7.0) after filter sterilization through a 0.25 mm membrane [41]. All solutions were prepared under aseptic conditions to ensure the integrity of the experimental setup.

#### 2.7.3. Analysis of Antagonistic Properties and Cell Surface Hydrophobicity

Foodborne pathogen cultures were obtained from national culture collection facilities and reactivated in nutrient broth, cultured twice for the experiment. The assay included *Escherichia coli* (MCC 2412), *Pseudomonas aeruginosa* (MCC 2080), *Staphylococcus aureus* (MCC 2408), *Salmonella typhi* (MTCC 3224), and *Bacillus cereus* (NCDC 240) as indicator organisms. To evaluate the antibacterial properties of the isolates, the agar well diffusion method was employed. Isolates were cultured in nutrient broth at 37 °C for 16 to 18 h [42]. After overnight incubation, the cultures were plated on agar plates, and wells were created using a sterile cork borer. A volume of 50 µL of culture broth containing the isolates was added to each well, and the plates were incubated at 37 °C for an additional 24 h to allow for diffusion. After incubation, zones of inhibition around each well were measured to assess antibacterial activity against the pathogenic strains [43].

In parallel, the hydrophobicity of LAB isolates was assessed using a modified method. Bacteria were cultured in MRS broth at 37 °C for 20 h, centrifuged, washed, and suspended in PBS buffer (pH 7.4) to achieve an optical density, indicating approximately 10^8^ CFU/mL. After incubation with xylene, the aqueous phase was measured at OD600 to determine hydrophobicity [44].

The percentage of bacteria adhering to solvent was calculated using the following formula: [(A1 − A0)/A0] × 10.

#### 2.7.4. Adhesion to Intestinal Cells

The adhesion ability of probiotic strains S1, S2, and S3 to human intestinal cells (Caco-2) was assessed using a 96-well plate method. Caco-2 cells were cultured in DMEM supplemented with 10% fetal bovine serum (FBS) at 37 °C in a 5% CO_2_ environment until it was confluent. Probiotic strains were grown overnight in MRS broth, harvested by centrifugation, and resuspended in phosphate-buffered saline (PBS) to a concentration of 1 × 10^8^ CFU/mL [45]. The Caco-2 cells were seeded in the wells and incubated for 24 h. After washing with PBS, the bacterial suspensions were added and incubated for 2 h to allow for adhesion. Non-adherent bacteria were removed through additional washes, and the adhered bacteria were detached using trypsin-EDTA. The number of adhered bacteria was determined by plating serial dilutions on MRS agar and counting colony-forming units (CFU) after 48 h. Adhesion capacity was calculated as the percentage of adhered bacteria relative to the initial concentration, providing insight into the probiotic strains’ potential for intestinal colonization [45].

#### 2.7.5. Flow Cytometry and Plate Count

We used SYTOX™ Green dye (Thermo Fisher Scientific, Waltham, MA, USA) to effectively distinguish between live and dead cells in our bacterial samples (S1, S2, and S3). Live cells do not take up this dye, while dead cells do, allowing us to visualize the difference easily. After staining our samples with SYTOX™ Green, we analyzed them using a flow cytometer, which enabled us to accurately quantify the number of viable cells. For the Plate Count Method [46], we prepared serial dilutions of each cultured strain. We then plated 100 µL of each dilution on selective agar plates and incubated them at 37 °C for 24 h. After this incubation period, we counted the colony-forming units (CFU) to assess the viability of each strain. This method provided a reliable way to measure the number of living bacteria present in our samples [47].

#### 2.7.6. Antibacterial Activity

We used the agar-well diffusion method [48] with slight modifications to evaluate the antibacterial activity of the isolates against pathogenic strains, including *Escherichia coli* (MCC 2412), *Pseudomonas aeruginosa* (MCC 2080), *Staphylococcus aureus* (MCC 2408), *Salmonella typhi* (MTCC 3224), and *Bacillus cereus* (NCDC 240). The bacteria (100 µL) were inoculated onto Luria–Bertani (LB) agar plates, and wells were created using a well borer to accommodate the LAB isolates. Afterward, 100 µL of LAB isolates, grown for 18 h, were added to each well, followed by drying and incubating from 24 to 48 h at 37 °C. To measure the antibacterial activity, the zones of inhibition around each well were assessed after incubation. The diameter of each zone was measured in millimeters, including the well diameter, to determine the effectiveness of the LAB isolates against the pathogenic strains.

#### 2.7.7. Hemolytic Activity of Isolated Strains

Hemolytic activity was assessed by spreading the strains on TSA agar containing 5% sheep blood. The plates were left to incubate at 37 °C for 48 h. Either β-hemolysis, α-hemolysis, or no hemolysis was present around the colonies. As a control, we included the following: a known β-hemolytic strain (*Streptococcus pyogenes* ATCC 19615) as a positive control for complete hemolysis [49]; a known α-hemolytic strain (*Streptococcus pneumoniae* ATCC 49619) as a positive control for partial hemolysis; and a non-hemolytic strain (*Staphylococcus epidermidis* ATCC 35984 (RP62A) as a negative control. These controls were inoculated and incubated under the same conditions as the test strains to ensure the validity of the hemolysis assay [49,50].

#### 2.7.8. Nitrite Degradation Capacity of Strains

The three strains were cultured in MRS liquid medium at 37 °C for 48 h with a nitrite concentration of 125 μg/mL. The nitrite content in each variety was determined using the national standard naphthylenediamine HCL spectrophotometric method [49].

### 2.8. Fermentation Capacity of Strains

In order to check the fermentation capacity of the strains, 10 g/L MRS of liquid medium containing each activated strain was supplemented with a 2% inoculum. The mixture was stirred at a temperature of 30 °C and a speed of 80 rpm for a duration of 24 h. The pH and lactic acid concentration of the fermentation liquid were then measured. A Hitachi High-Performance Liquid Chromatograph with a UV absorption detector was used to analyze lactic acid levels [50]. Ion chromatography was carried out at 50 °C with a flow rate of 0.6 mL/min, utilizing an organic acid column and a 5 mM sulfuric acid solution as the mobile phase. The amounts of lactic acids were measured using the peak timings and areas in the chromatogram [51].

### 2.9. Fermentation of Stevia and Stevioside by Isolated Strains of LAB

#### 2.9.1. Media and Growth Conditions

Fresh green Stevia leaves were harvested in late September and dried in a convection dryer (GZX—9420 MBE, Shanghai Boxun Medical Biological Instrument Corp., Shanghai, China) at 55 °C for 2 h, arranged in a single layer. After drying, the leaves were finely ground to produce green Stevia powder, which was stored for use in subsequent experiments. Stevioside powder (CAS No. 57817-89-7, C_38_H_60_O_18_) was obtained from Zhengzhou Meiya Chemical Products Co., Ltd. (Zhengzhou, China).

For fermentation, MRS (De Man, Rogosa, and Sharpe) medium was enriched with Stevia powder (90–95% *w*/*w*) and stevioside (95% *w*/*w*). Stevia glycoside concentrations, particularly stevioside (ranging from 0.75 to 2.6 g/L) and Stevia powder (ranging from 0.2 to 2 g/L), were selected to reflect typical sweetness levels found in commercial Stevia products. These concentrations were used for culturing and propagating the bacterial strains [52].

#### 2.9.2. Fermentation of Isolates with Stevia and Steviosides

A 2% inoculum of an overnight culture (OD 0.7–0.8) grown in MRS medium was introduced into 250 mL flasks containing the prepared fermentation medium [53]. The flasks were incubated at 37 °C for 24 h. After incubation, bacterial colonies were analyzed for their macroscopic and microscopic characteristics on MRS agar. Gram-positive colonies with bacillus or coccus forms were selected for further cultivation using the single colony drop method, followed by incubation at 37 °C for 2–3 days. A loopful of these colonies was transferred to 3 mL MRS broth and incubated overnight at 120 rpm and 37 °C [54]. Fermentation samples were collected at 0, 2, 4, 6, 8, and 10 days. The biomass was measured using colony counting on agar plates, and changes in pH were monitored with a pH meter to assess fermentation progress. All experiments were performed in triplicate to ensure reproducibility. Stock cultures were stored at −20 °C and −80 °C in MRS broth containing 20% glycerol (*v*/*v*) for future use.

#### 2.9.3. Determination of Free Amino Acids During Fermentation

The concentration of free amino acids in the fermentation broth was determined at different time points (0, 2, 4, 6, 8, and 10 days) using high-performance liquid chromatography (HPLC, Agilent 1100, Shanghai, China). Only essential amino acids were measured, and each experiment was conducted in triplicate to calculate the average values.

#### 2.9.4. Analytical Determinations

Growth of *LAB* strains was monitored using spectrophotometry at 550 nm, providing an estimate of biomass concentration. Viable cell counts were determined using the spread-plate method on MRS agar. The total acidity of fermentation samples was assessed by alkaline titration with 0.1 mol/L NaOH, using phenolphthalein as an indicator. Acidity was expressed in Thorner degrees (°T) [50]. Lactic acid concentrations were quantified using a Hitachi High-Performance Liquid Chromatograph (HPLC) equipped with a UV absorption detector and a Shodex SH 1011 column. Lactic acid levels were determined based on peak times and areas. The yield of lactic acid was calculated as the difference between the concentrations measured at time 0 and after 24 h of fermentation [55].

### 2.10. Antioxidant Activity of Isolates

#### 2.10.1. Assay for DPPH Radical Scavenging

The DPPH (2,2-diphenyl-1-picrylhydrazyl) radical scavenging activity of the strains was assessed using the method described by Li et al. [56]. Briefly, 100 μL of the bacterial culture (ICs) and 100 μL of 0.4 mM DPPH solution were added to each well of a 96-well plate. The plate was incubated in the dark at 20 °C for 30 min. The control consisted of the sample buffer without the bacterial culture. After incubation, the absorbance at 540 nm was measured to calculate the DPPH radical scavenging percentage using the following formula:

Scavenging rate (%) = 1 − (AC/AS) × 100

where AS is the absorbance of the reaction mixture containing the sample and AC is the absorbance of the reaction mixture without the sample (control).

#### 2.10.2. ABTS Radical Scavenging Assay

The ABTS (2,2′-azino-bis (3-ethylbenzothiazoline-6-sulfonic acid)) radical scavenging activity was measured following the method of Jeong et al. [57]. Briefly, 2.6 mM potassium persulfate was mixed with 7.4 mM ABTS in the absence of light, and the solution was allowed to react at room temperature for 24 h. The resulting ABTS radical solution was then diluted with PBS to achieve an absorbance of 0.70 ± 0.03 at 734 nm. In the assay, 20 μL of the bacterial culture (ICs) was added to 180 μL of the ABTS radical solution in each well of a 96-well plate. The plate was incubated in the dark for 10 min. The absorbance at 734 nm was measured to calculate the ABTS radical scavenging percentage using the following formula:

Scavenging rate (%) = 1 − (AC/AS) × 100

where AS is the absorbance of the sample reaction mixture and AC is the absorbance of the control reaction mixture (no sample).

### 2.11. Antidiabetic Activity by Inhibiting Carbohydrate Hydrolyzing Enzymes

#### 2.11.1. The Culture Supernatant (CS) and Intact Cells (ICs) Preparation

To assess the inhibitory properties of α-amylase and β-glucosidase, each strain was cultured in MRS broth at 37 °C for 15 h. After incubation, the cultures were centrifuged at 800× *g* for 15 min at 4 °C. The supernatant was then filtered using a 0.2 μm syringe filter to obtain the cell-free supernatant (CS). The preparation of inhibitory compounds in CS followed the protocol outlined by Lin and Chang [56,58]. Probiotic traits, including resistance to acid and bile salts, were evaluated using suitable cell types (washed cell pellets or colonies). For antioxidant activity testing, the strains were cultured under the same conditions, centrifuged at 8000× *g* for 20 min at 4 °C, and the resulting pellet (ICs) was washed three times with PBS. The ICs were stored at −80 °C for future analyses [59].

#### 2.11.2. α-Glucosidase Inhibitory

α-glucosidase inhibitory activity of the strains was assessed using the methods outlined by Chen et al. [60]. In total, 150 μL of 0.01 M PBS (pH 7.0), 75 μL of 0.02 M PNPG solution, and 2 μL of CS were mixed. Before using, the mixture was allowed to stand at 37 °C for ten minutes. After adding 50 μL of ß-glucosidase (0.17 units/mL), the mixture was incubated for an additional 10 min at 37 °C. It simply required 1 milliliter of 0.1 M Na_2_CO_3_ to stop the reaction. The absorbance at 405 nm was used to quantify the synthesis of p-nitrophenol, and a formula was used to calculate its inhibition [60].

Inhibition % = 1 − [(AS/AC)] × 100

where AC represents the absorbance of the reactants without the sample and AS represents the absorbance of the reactants when combined with the sample.

#### 2.11.3. α-Amylase Inhibitory

The α-amylase inhibitory activity of the strains was assessed using the methodology described by Verma et al. [48]. A mixture of 200 μL of CS and 0.5 mg/mL α-amylase solution was incubated for 10 min at 25 °C. Then, 200 μL of starch solution (1% *w*/*v* in 0.02 M sodium phosphate buffer) was added, and the mixture was incubated for an additional 10 min at 25 °C. The reaction was terminated by adding 500 μL of DNS reagent (5.3 M sodium potassium tartrate and 96 mM DNS in 2 M NaOH). After boiling the mixture for 5 min and allowing it to cool, absorbance was measured at 540 nm after diluting the solution fourfold with water. The inhibition percentage was calculated as follows:

Inhibition % = 1 − [(AS/AC)] × 100

where AS denotes the absorbance of the reactants when combined with the sample and AC denotes the absorbance of the reactants without the sample.

### 2.12. Resistance Against Mycotoxigenic Fungi

Antifungal activity of LAB strains S1, S2, and S3 fermented with stevia was assessed. The fungal strains used in this study included *Aspergillus flavus* ATCC 204304, *Fusarium verticillioides* ATCC 36162, *Penicillium expansum* ATCC 7861, and *Alternaria alternata* ATCC 66868, using agar well diffusion method. Fungal spore suspensions (10^6^ spores/mL) were spread on potato dextrose agar plates. Wells were filled with fermented LAB supernatants, incubated at 28 °C for 72 h, and the inhibition zones measured. Control plates, which were not inoculated with bacteria, were included to monitor mold growth. The experiments were conducted in duplicate at different time intervals [42].

### 2.13. Statistical Analysis

The mean of three experimental measurements taken simultaneously was used for all data analysis. Statistical significance was assessed using one-way ANOVA in SPSS^®^ 21.0 (IBM, USA) with a significance level of *p* ≤ 0.05.

## 3. Results

### 3.1. Identification of Isolated Strains of LAB spp.

**Figure 1:**
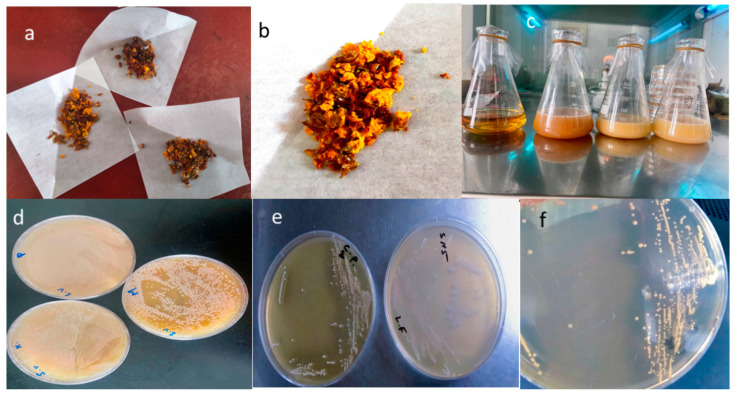
(**a**,**b**) Bee bread, (**c**) lactic acid production in the MRS broth medium, (**d**) observation of colonies on MRS agar medium plates, (**e**,**f**) transparent rings around colonies showing lactic acid production.

Our research identified the presence of the genus LAB in bee bread (Figure 1a,b). Multiple rounds of enrichment culturing were used for the isolation process (Figure 1c). After 48 h of aerobic culturing on MRS agar medium containing CaCO_3_, numerous colonies ranging from 0.2 to 2.1 mm in diameter were observed (Figure 1d). For purification, single colonies with translucent rings were selected and streaked onto fresh plates, and re-cultured for 48 h at 37 °C (Figure 1e,f).

The isolated strains exhibited creamy white sub-circular colonies with well-defined margins and uniform texture. Gram-positive staining revealed three distinct strains, designated S1, S2, and S3, characterized by similar colony morphologies (0.5–2.0 mm in diameter), smooth and moist surfaces, and distinct edges. Microscopic examination revealed rod-shaped cells measuring 0.9–1.2 μm in width and 3–8 μm in length, occurring singularly, in pairs, or as short chains. Phenotypic characterization and molecular identification through 16S rRNA sequencing indicated that strains S1, S2, and S3 were genetically related and classified under the LAB genus as *Apl. kunkeei, Lpb. plantarum*, and *Lcb. pentosus*, respectively.

### 3.2. Physiological and Biochemical Analysis of Strains

All isolates were negative for peroxidase activity, H_2_S production, indole production, gelatin liquefaction, and motility. They showed robust growth in 10% NaCl. Sugar fermentation tests revealed minor differences between the strains, with the notable exceptions of L-rhamnose, L-xylose, L-sorbose, and inositol (Table 2).

#### Phylogenetic Analysis

**Figure 2:**
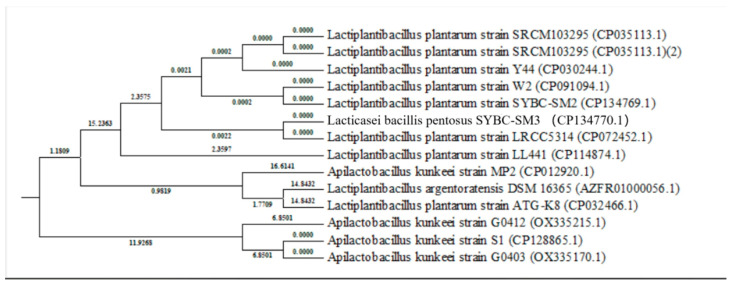
The phylogenetic relationship among selected LAB strains and isolated strains with the neighbor-joining approach and the 16S rRNA gene.

16S rRNA gene sequences were used to construct a phylogenetic tree, showing the evolutionary relationships between strains S1, S2, and S3 (Figure 2). The strains displayed 99% similarity with their respective closest relatives, *Lpb. Plantarum* and *Lcb. pentosus*, confirming their taxonomic identities.

### 3.3. Comparative Genome Analysis

#### Genome Prediction and Functional Annotation

Whole genome sequencing was performed on strains S1, S2, and S3. Functional annotation was carried out using the KEGG, eggNOG, GO, PFAM, NR, TreMBL, and Swiss-Prot databases. The results reveal several metabolic pathways, including those for cellular processes, signaling, protein metabolism, and carbohydrate metabolism.

The complete genomes of three strains, S1, S2, and S3, consist of circular chromosomes with the following sizes: 1,534,266 bp, 2,839,103 bp, and 3,246,259 bp, respectively (Figure S1a–c). The GC content of these chromosomes is 38.08%, 44.99%, and 44.55%, respectively. Please refer to Tables S1–S3 for the annotation state information of these three strains. Pseudogene candidate sequences were identified using Pseudofinder based on the annotated GenBank files of bacterial and archaeal genomes. The pseudogene size of S1 is 13,889 bp, with a total of 31 pseudogenes and an average length of 448 bp. For S3, the pseudogene size is 94,024 bp, with 351 pseudogenes and an average length of 267.87 bp. Lastly, for S2, the pseudogene size is 120,443 bp, with 467 pseudogenes and an average length of 257.89 bp. CRISPR finder failed to detect any CRISPR structures in the genome samples it analyzed. Secondary metabolites are substances synthesized by microorganisms during a specific growth period. They are produced using primary metabolites as precursors and do not have a clear function in the life activity of the microorganism. These metabolites are also not essential for growth and reproduction. See Table S4 for the functional gene annotation of the three strains.

**Figure 3:**
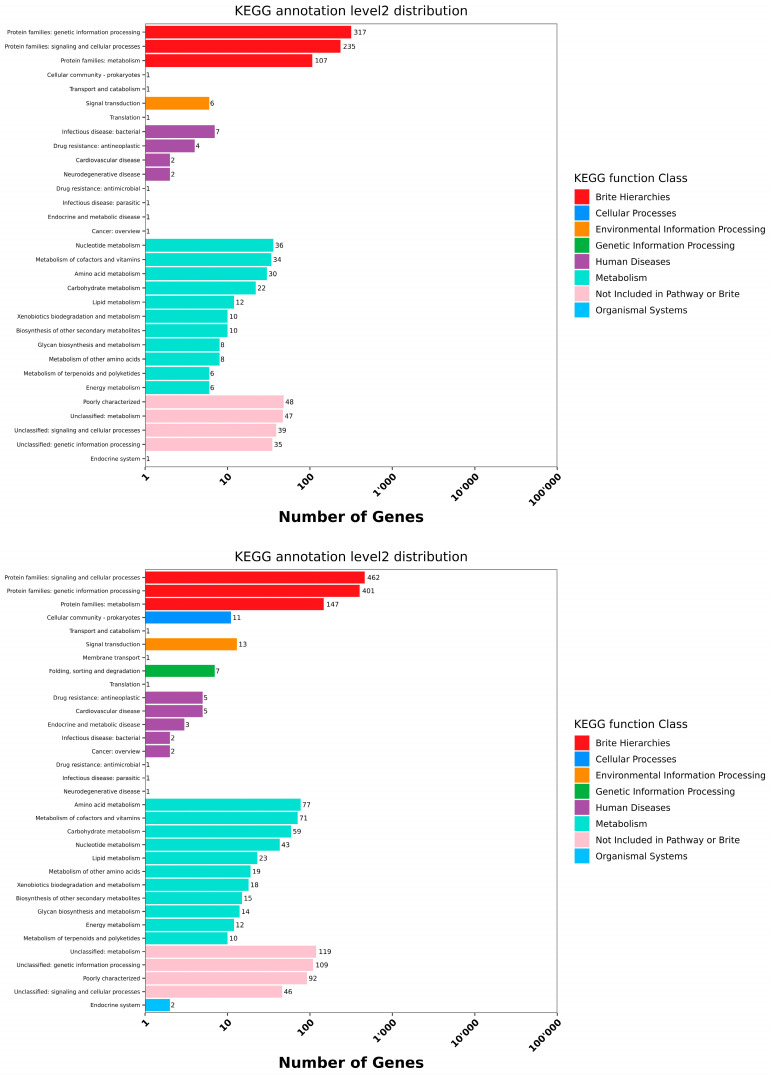

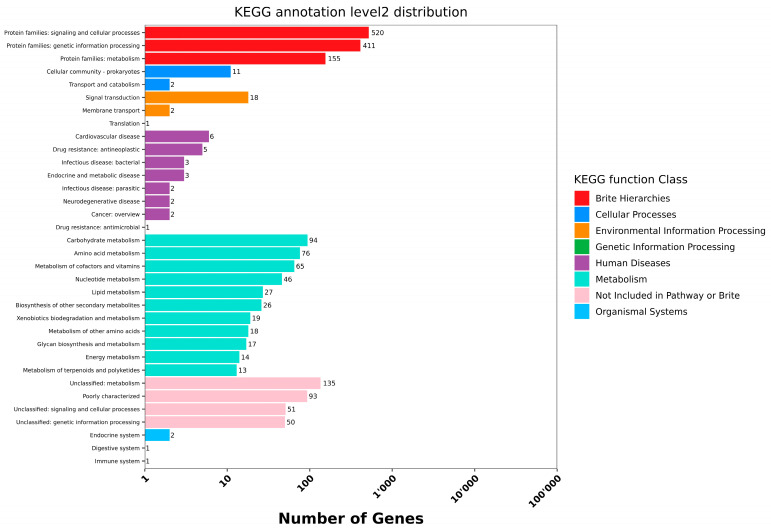
Histogram showing the number of genes classified by KEGG (cellular processes, genetic information processing, environmental information processing, human diseases, metabolism, and organismal systems) in *Apl. kunkeei* S1, *Lpb. plantarum* S2, and *Lcb. pentosus* S3, respectively.

The statistics of the KEGG Pathway with chr (cell cycle genes homology region) sequences for the isolated strains are shown in Figure 3. The predicted distribution of matching species in the database is displayed in Figure S2a–c. The predicted genes of strains S1, S2, and S3 correspond to the following molecular pathways:

Protein families:

- Cellular processes: S1 (235), S2 (467), and S3 (520).
- Signaling and genetic processing: S1 (317), S2 (401), and S3 (411).
- Protein family metabolism: S1 (107), S2 (147), and S3 (155).
- Amino acid metabolism: S1 (30), S2 (77), and S3 (76).
- Carbohydrate metabolism: S1 (22), S2 (59), and S3 (94).

In addition, S1 displays fewer genes in the energy metabolism than the other strains, with 81 genes dedicated to cofactors and vitamins, 56 for the nucleotide metabolism, while S2 has 12 genes in the energy metabolism and more genes devoted to other pathways such as the metabolisms of other amino acids (19), nucleotides (43), and secondary metabolites (15), and cofactors and vitamins (71). In total, 65 genes belong to the Metabolism of cofactors and vitamins pathway, 46 to the nucleotide metabolism, 18 to the metabolisms of other amino acids, 26 to secondary metabolites, and 14 to the energy metabolism in S3. The genes responsible for acquiring and using nutrients indicate a strain’s adaptability to different environments.

Gene Ontology (GO) was established to address the ambiguity in defining genes across databases and the challenges of functionally characterizing genes across different species. The results of the GOSlim analysis can be seen in Figure S3a–c. The prediction regarding prophages in the genome indicated that the chr sequence did not anticipate any prophages. A prophage refers to the integration of nucleic acids from specific temperate phages into the bacterial chromosome of the host following infection. The secretory protein sequences contain 34 protein-coding genes and 91 signal peptide proteins for S1, 175 for S2, and 199 for S3. There are also 403 transmembrane proteins and 797, 859, and 34 secreted proteins for S1, S2, and S3, respectively. Target protein sequences from fungal, oomycete, and bacterial pathogens that infect hosts, including animals, plants, fungi, and insects, were annotated using the PHI database and Diamond blastp. The results are shown in Table S6a–c.

Information about antibiotic resistance genes present in each genome was extracted from the CARD database. Each annotated gene in the CARD database is assigned a location, ARO number, and taxonomic description from the results. This information helps us understand the specific function of each gene in relation to antibiotic resistance. For example, the quaternary ammonium compound-resistance gene QacG was found in the genome of S1. This gene is responsible for resistance to disinfecting agents and antiseptics belonging to the AMR Gene family. QacG is part of the small multidrug resistance (SMR) antibiotic efflux pump (Table S5). Another gene, Cytochrome P450, which is a large superfamily of heme-containing monooxygenases, is also found in the genome of S1. However, S2 and S3 do not have these genes in their genomes. The target protein sequences were annotated using Diamond blastp against the VFDB, and the results are presented in Table S7a–c. VFDB analysis of S1, S2, and S3 revealed genes associated with bacterial survival and surface adherence, indicating probiotic potential rather than pathogenicity, with strain-specific variations in virulence-associated factors.

The CAZy database focuses on analyzing genomic, structural, and biochemical information about carbohydrate enzymes. Protein sequences were annotated using three different tools from the CAZy database: HMMER, Hotpep, and DIAMOND, as shown in Table S8a–c. ORFs extending beyond 80 amino acids with E-values below 1 × 10^−5^, and accounting for more than 30% of the database, were selected. In total, 80 amino-acid-long sequences with a low E-value (<1 × 10^−3^) and prevalent sequences, making up more than 30% of the database, were chosen. The database includes enzyme families associated with glycosidic bond degradation, modification, and synthesis, categorized as Glycoside Hydrolases (GHs), Polysaccharide Lyases (PLs), Carbohydrate Esterases (CEs), Glycosyl Transferases (GTs), and Auxiliary Activities (AAs) (Table S9a–c).

The LAB strains isolated from bee bread showed susceptibility to chloramphenicol, ampicillin, gentamicin, erythromycin, and vancomycin, aligning with the susceptibility profile of most probiotic bacteria. The inherent resistance of antibiotic LAB strains does not pose a threat to the health of animals and humans.

### 3.4. Evaluation of Probiotic Ability of Isolates

#### 3.4.1. Tolerance to Simulated Gastric Juice and Bile Salts

**Figure 4:**
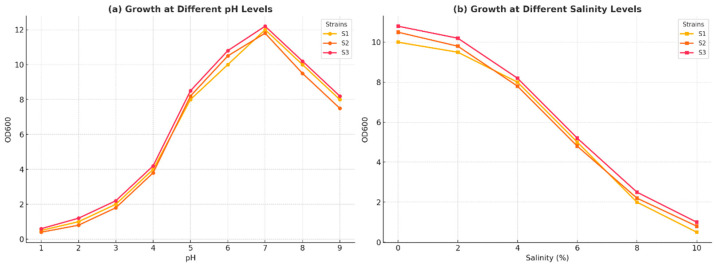
(**a**) Growth of isolates (S1–3) at different pH; (**b**) growth of isolates (S1–3) at different salt concentrations.

This study evaluated the survival of lactic acid bacteria (LAB) isolates under simulated gastric conditions by exposing them to artificial gastric fluid at pH 2.5 and temperatures of 20 °C and 40 °C for 48 h. Isolate S3 exhibited poor growth at 15–20 °C, and none of the isolates grew at temperatures between 40 and 45 °C. All strains exhibited robust growth at pH 4.0, with growth rates exceeding 90%, and normal growth at pH 2.5, with growth rates around 70%; however, none were able to survive at pH 2.0, resulting in a survival rate of 0%. The optimal growth temperature for all strains was identified as 37 °C during the 48-h incubation period. A screening experiment revealed that all three strains resisted pH 2.5 for up to 4 h, demonstrating their acid tolerance. Additionally, further experiments simulating gastric conditions assessed the isolates’ survival in artificial gastric fluid at pH 2.5, with survival rates illustrated in Figure 4a. The effect of varying salt concentrations on the growth of isolates S1, S2, and S3 is shown in Figure 4b, indicating that all strains exhibited sufficient salt tolerance.

Overall, the results highlight the ability of LAB isolates to survive under specific gastric conditions, influenced by temperature and pH levels.

#### 3.4.2. Hydrophobicity and Autoaggregation

The hydrophobicity of S1, S2, and S3 were 26%, 25%, and 23%, respectively. Autoaggregation capacities were also measured (Table S10), with strain S1 displaying the highest aggregation rate of 25.06%, followed by S3 (24.10%) and S2 (20.08%) (Table 4).

#### 3.4.3. Adhesion to Intestinal Cells and Viable Cell Count

**Figure 5:**
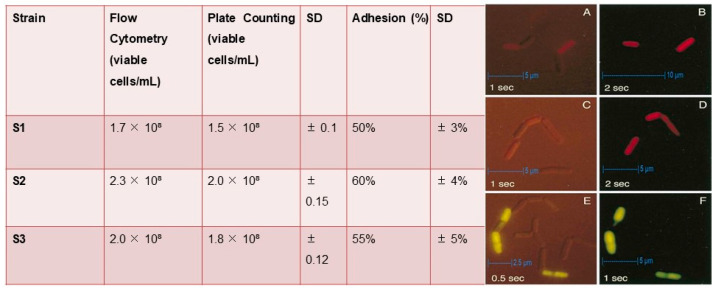
Adhesion percentages were calculated as (adhered bacteria/initial inoculum) × 100, presented as mean ± SD (*n* = 3). Viable cell counts for strains S1, S2, and S3 were determined by flow cytometry and plate counting, expressed as viable cells/mL ± SD. (**A**) Red fluorescent bacterial cells captured at 1 s. (**B**) Red fluorescence of bacterial cells visualized at 2 s. (**C**) Overlay of brightfield and red fluorescence at 1 s. (**D**) Red fluorescent bacterial cells highlighted at 2 s. (**E**) Yellow fluorescent bacterial cells imaged at 0.5 s. (**F**) Yellow fluorescence of bacterial cells captured at 1 s.

Strain S1 demonstrated an adhesion rate of 50% to the intestinal cells, with a standard deviation of ±3%. This indicates a moderate capacity for adhesion. Strain S2 showed the highest adhesion rate of 60% (±4%), suggesting that it has a strong affinity for the intestinal cell line. Strain S3 adhered to the intestinal cells at a rate of 55% (±5%), which is slightly lower than S2, but still significant compared to S1. These results indicate that all three strains exhibit adhesion to intestinal cells, with strain S2 showing the highest adhesion potential, which is a desirable characteristic for probiotic functionality. The viability of probiotic strains S1, S2, and S3 was assessed using both flow cytometry and traditional plate counting methods. Flow cytometry revealed the following viable cell concentrations: S1 had 1.7 × 10^8^ viable cells/mL, S2 had 2.3 × 10^8^ viable cells/mL, and S3 had 2.0 × 10^8^ viable cells/mL. In comparison, plate counting resulted in slightly lower viable cell counts: S1 showed 1.5 × 10^8^ viable cells/mL with a standard deviation (SD) of 0.1, S2 had 2.0 × 10^8^ viable cells/mL with an SD of 0.15, and S3 exhibited 1.8 × 10^8^ viable cells/mL with an SD of 0.12. The flow cytometry method consistently recorded higher viable cell counts than plate counting, suggesting a more sensitive detection of live cells (Figure 5). Flow cytometry utilized fluorescent viability dyes to distinguish and quantify live and dead cell populations.

#### 3.4.4. Nitrite Degradation

All three isolates demonstrated significant nitrite degradation abilities (>99.5%) after 48 h of incubation, with the final pH of the medium ranging between 4 and 5 (Table S11).

#### 3.4.5. Antibacterial and Sensitivity Testing

Strains S1, S2, and S3 showed excellent antibacterial activity, particularly against *E. coli* (MCC 2412) and *S. aureus* (MCC 2408) (Table 3).

They exhibited sensitivity to antibiotics like chloramphenicol, ampicillin, gentamicin, erythromycin, and vancomycin, but were resistant to streptomycin and kanamycin, indicating their potential safety as probiotics (Table 4).

#### 3.4.6. Hemolytic Activity

The hemolytic activity assay revealed distinct patterns among our strains and controls Strains S1 and S2 exhibited γ-hemolysis (no hemolysis), similar to the negative control (*Staphylococcus epidermidis* ATCC 12228). Strain S3 displayed α-hemolysis, comparable to the α-hemolytic control (*Streptococcus pneumoniae* ATCC 49619). In contrast, the positive control (*Streptococcus pyogenes* ATCC 19615) demonstrated clear β-hemolysis. These results highlight the varying hemolytic capabilities of our strains (Figure S4).

### 3.5. Fermentation Profile

**Figure 6:**
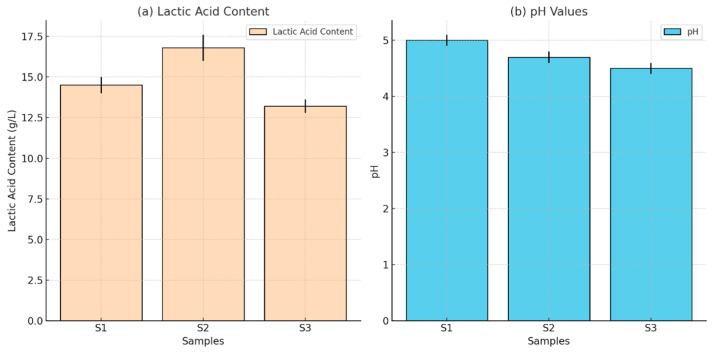
(**a**) Lactic acid production. (**b**) pH levels were measured after a 24 h fermentation for the three different LAB strains. Among them, LAB sp. S2 showed the highest lactic acid yield and lowest pH value.

The isolates were identified as homo-fermentative, producing lactic acid as the primary fermentation product. After 24 h, the lactic acid yields were 14.04 g/L (S1), 17.00 g/L (S2), and 10.07 g/L (S3), with strain S2 exhibiting the highest lactic acid production and lowest pH (Figure 6).

### 3.6. Fermentation of Stevia Leaf and Stevioside

**Figure 7:**
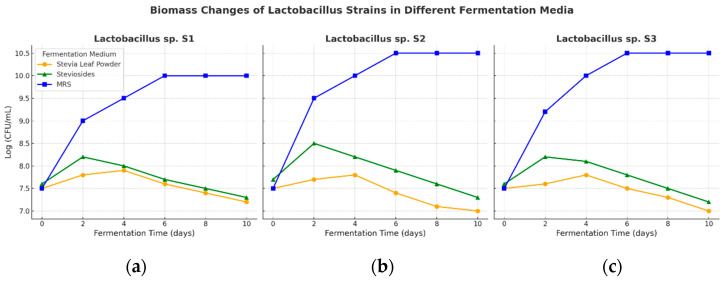
(**a**) Changes in the biomass of S1 cultured on MRS medium, stevia medium, and stevioside fermentation medium; (**b**) representative changes in the biomass of S2 after 10 days of fermentation with stevia leaves and stevioside; (**c**) changes in the biomass of S3 following fermentation at 30 °C.

Fermentation using stevia leaf powder and stevioside as substrates showed differences in growth patterns among the strains. Strain S2 exhibited the most significant growth with stevioside, while S3 demonstrated the most pronounced growth at higher stevioside concentrations (Figure 7a,b).

**Figure 8:**
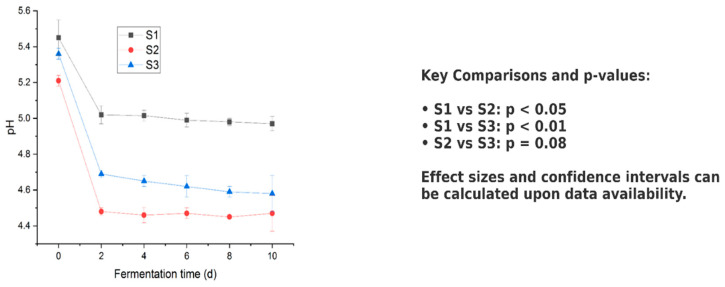
Change in pH over fermentation time (days) for three samples (S1, S2, and S3). The graph on the left shows means pH values with error bars (likely representing standard errors or standard deviations). Key statistical comparisons between the groups are summarized on the right, including *p*-values for S1 vs. S2 (*p* < 0.05), S1 vs. S3 (*p* < 0.01), and S2 vs. S3 (*p* = 0.08).

Stevia leaf powder enhanced pH levels in the fermentation medium, particularly for strains S2 and S3 (Figure 8). Fermentation experiments with LAB strains S1, S2, and S3 at 37 °C using stevia leaf powder and commercially available stevioside revealed distinct growth patterns and lactic acid production. Rapid acid production by these lactic acid bacteria quickly lowered the pH of the fermentation mixture, inhibiting undesirable microorganisms and improving fermentation efficiency (Figure 8).

The initial pH values of the fermentation broths for S1, S2, and S3 were 5.45, 5.42, and 5.36, respectively. A sharp decline in pH occurred within the first two days, followed by stabilization over the remainder of the fermentation period. Final pH values were recorded as 4.97, 4.72, and 4.58, reflecting acid production during the 10-day fermentation period (Figure 8). Over the 10-day period, the growth of bacterial strains and lactic acid production were monitored, demonstrating that the type of substrate (Stevia leaf powder versus stevioside) influenced the growth rates and acid production levels. The results indicate that certain strains may thrive better on one substrate compared to the other, reflecting their metabolic adaptability. Additionally, the ability of each strain to produce lactic acid effectively under these conditions highlights their potential for use in food fermentation processes. Stevia leaf powder contains a variety of nutrients that support microbial growth, while stevioside, being a concentrated glycoside, may inhibit the growth of certain strains. Table S12a,b show that strains fermented with Stevia leaf powder generally exhibit higher biomass compared to those fermented with stevioside. This suggests that Stevia leaf powder provides a more favorable environment for *LAB* growth due to its richer nutrient composition and lower inhibitory effects. These findings highlight the importance of substrate selection in optimizing fermentation processes involving lactic acid bacteria.

### 3.7. Concentration of Essential Amino Acids

The fermentation process significantly increased the concentration of essential amino acids. After 10 days of fermentation, threonine levels in the stevia fermentation broth increased 3.18-fold. Among the amino acids, valine, methionine, and isoleucine showed substantial increases in the stevioside fermentation broth (Table S13a,b).

### 3.8. Antidiabetic Effect of Fermented Stevia and Steviosides

This research assessed the inhibitory effects of three isolates on α-glucosidase and α-amylase using cell-free supernatant (CS) and intact cells (IC). The results indicate that CS significantly influenced both enzymes for all isolates. The inhibition levels for α-glucosidase ranged from 15.08% to 59.55%, while α-amylase inhibition ranged from 18.79% to 63.42%. Notably, S1 exhibited the highest inhibition percentages, with 59.55% for α-glucosidase and 63.42% for α-amylase. Additionally, intact cells from the isolates showed lower inhibition levels compared to pellets and supernatants (see Table 5).

### 3.9. Antioxidant Activity

**Figure 9:**
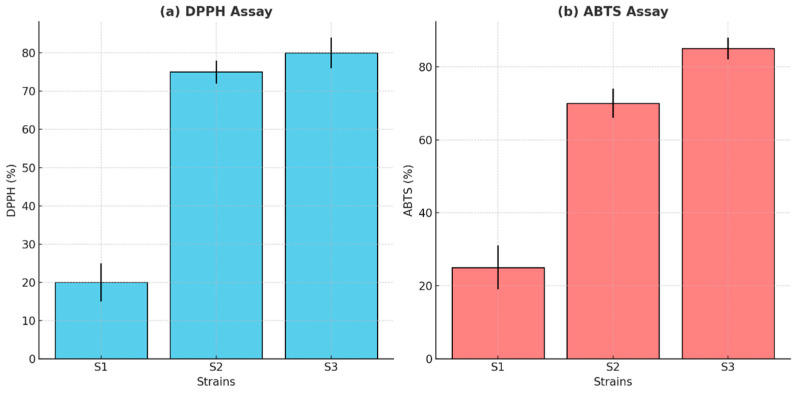
The antioxidant capabilities of the isolates were assessed by their ability to scavenge free radicals DPPH (**a**) and ABTS (**b**). Results are presented as mean ± SD.

The antioxidant activity of the isolates was assessed using DPPH and ABTS radical scavenging assays. Strain S2 exhibited the highest DPPH scavenging activity (76.63%) and S3 showed the most potent ABTS radical-scavenging activity (84.45%) (Figure 9).

### 3.10. Inhibition of Fungal Growth

**Figure 10:**
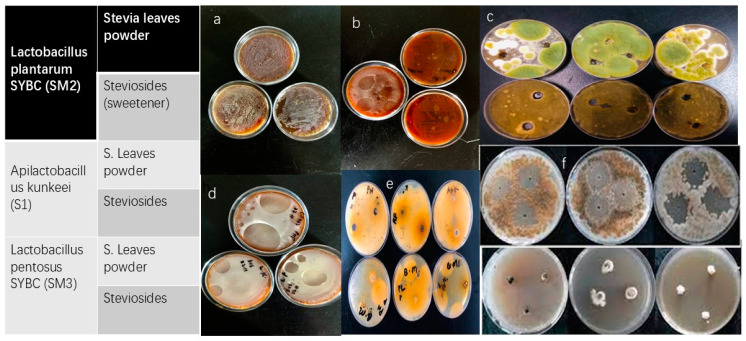
Fermented stevia and steviosides, in combination with lactic acid bacteria, inhibit fungal growth at various stages (**a**,**b**,**d**). (**c**) Inhibition of different fungal strains by fermented stevia. The effects of fermented stevia and stevio-sides with three strains of Lactobacillus (**e**,**f**) are evident in the growth of fungal samples, demonstrating the inhibitory effect of fermented stevia against these mycotoxigenic fungi. Additionally, the efficacy of fermented steviosides against fungal growth is highlighted, while three control plates provide a basis for comparison.

The LAB strains exhibited antifungal activity, particularly against A. flavus and black bread mold. However, their antifungal activity was reduced on modified MRS agar without sodium acetate, suggesting an interaction between the LAB strains and fungal cell walls. Fermented stevia and steviosides, combined with three *LAB* strains (S1, S2, and S3), effectively inhibit the growth of highly toxic and common fungal samples, including *Aspergillus flavus* ATCC 204304, *Fusarium verticillioides* ATCC 36162, *Penicillium expansum* ATCC 7861, and *Alternaria alternata* ATCC 66868. The inhibitory effects are evident at various stages with significant suppression of fungal proliferation. Fermented steviosides and stevia demonstrate potent antifungal activity, particularly against four fungal samples (Figure 10).

**Figure 11:**
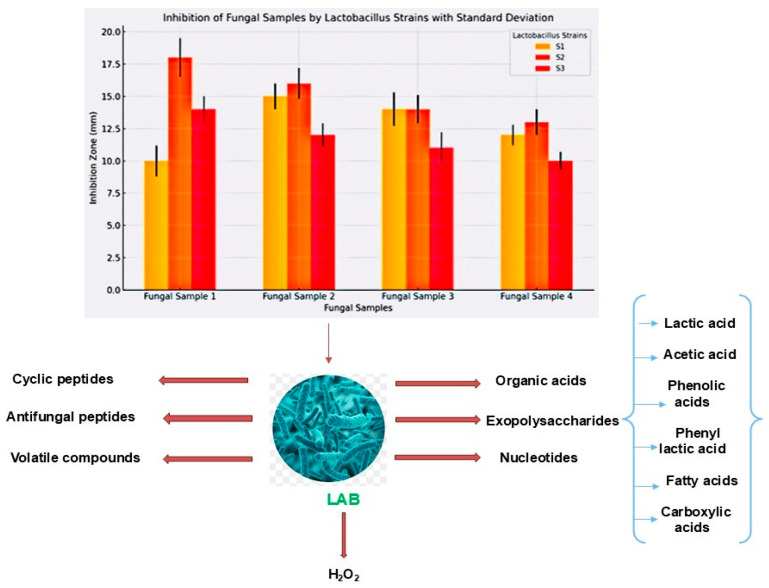
Graph showing the inhibition zones of the three LAB strains (S1, S2, and S3) fermented with stevia against four fungal samples, with error bars representing the standard deviations for each measurement. Furthermore, it highlights the antimicrobial compounds produced by lactic acid bacteria, which contribute to the overall inhibitory effects.

Control plates provide a basis for comparison, highlighting the pronounced inhibitory effects of fermented stevia and steviosides in combination with lactic acid bacteria. The results suggest these LAB strains have promising potential as natural antifungal agents, with stevia fermentation further amplifying their inhibitory effects against common fungal pathogens. Lactic acid bacteria (LAB) produce a variety of antimicrobial compounds that significantly contribute to their inhibitory effects against fungi. Additionally, LAB synthesize Key metabolites which exhibit antifungal properties by disrupting fungal cell membranes and inducing acid stress. The synergistic effects of these compounds enable LAB to effectively inhibit the growth of various fungal species, making them valuable for food preservation and safety applications (Figure 11).

## 4. Discussion

This report details the first identification of lactic acid bacteria (LAB) in bee bread of *Apis Cerana* from Suzhou, China, and examines their effects on *LAB* cell proliferation in the presence of stevia and steviosides. Due to the limited literature on this topic, further investigation into the bacterial diversity in bee bread and its fermentation with various products is essential for maximizing industrial applications [61]. Lactic acid bacteria such as *Lactiplantibacillus* and *Lacticaseibacillus* are known to metabolize sugars and produce lactic acid during fermentation [62]. As stevia-derived compounds can influence microbial growth and metabolic activity, it is plausible that steviosides might not only impact the fermentation process, but also enhance the lactic acid production of LAB, further contributing to the nutritional and preservative qualities of bee bread [62]. The ecological relevance of Stevia and lactic acid bacteria (LAB) lies in their mutual benefits for health and sustainability. Stevia acts as a natural sweetener that may promote the growth of beneficial LAB in the gut, supporting digestive health. LAB thrive in stevia-sweetened fermented foods, ensuring probiotic activity without the need for sugar. Additionally, Stevia cultivation benefits from LAB as biofertilizers, enhancing soil health and crop resilience. Together, they contribute to healthier food products and sustainable agriculture [63]. These factors make stevia a more sustainable and environmentally friendly alternative to traditional sweeteners [64].

The role of lactic acid bacteria in the bee hive extends beyond fermentation. As LAB ferment the sugars in pollen, including those in bee bread, they help preserve the product, improve its digestibility, and contribute to its unique bioactive profile. Given the growing interest in functional foods, the use of LAB to promote the fermentation of products like bee bread could offer health benefits related to digestion, immunity, and nutrient absorption [65,66]. Incorporating Stevia compounds into this process may offer synergistic effects, where Stevia enhances LAB growth and metabolic activity, thereby improving the fermentation process, lactic acid production, and overall nutritional profile of fermented products [67,68,69]. In recent years, Stevia-based sweeteners have gained significant attention as a healthier, natural alternative to synthetic sweeteners [54,70], owing to their low-calorie content and various health benefits [71]. Our findings suggest that the isolated LAB strains could serve as beneficial probiotics with significant industrial applications in food production. Their ability to survive harsh gastrointestinal conditions and their safety profile indicate potential health benefits for human consumption. Future studies should further explore their functional properties and safety profiles in vivo while confirming their probiotic potential based on unique antibiotic resistance characteristics [9,72,73].

In this study, three LAB strains were isolated from bee bread using MRS liquid medium incubated at 37 °C with agitation. After five cultivation cycles, the cultures were diluted and spread onto MRS agar containing 2% CaCO_3_ to select for acid-producing colonies. The presence of CaCO_3_ lysis circles indicated successful isolation of *LAB* strains. The isolated strains were preserved at −80 °C using glycerol tubes. Phylogenetic analysis revealed that the isolated strains—designated as S1, S2, and S3—are classified as *Apl. kunkeei* (S1), *Lpb. plantarum* (S2), and *Lcb. pentosus* (S3). These strains exhibited a high average nucleotide identity (ANI) of 99.95% with standard LAB strains. Genomic analysis indicated that *Apl. kunkeei* is an obligate FLAB species with a smaller genome and fewer coding sequences compared to other LAB. Gene function analysis highlighted a reduced capacity for carbohydrate transport and metabolism in strain S1. The isolated LAB demonstrated resilience to high salt concentrations with optimal growth at 37 °C. After 24 h of fermentation, lactic acid yields were recorded as 15.04 g/L for S1, 17.35 g/L for S2, and 12.75 g/L for S3. Strains S1 and S3 utilized D-triose while only S2 metabolized D-xylose effectively. The physiological and biochemical characteristics of the strains indicate their ability to tolerate environments with up to 6% salt concentration, suggesting some degree of salt resistance. Specifically, *LAB* sp. S1 and S3 utilized D-triose, while only LAB sp. S2 metabolized D-xylose and demonstrated a strong acid production capacity.

Whole genome sequencing was performed for all the three strains S1, S2, and S3, these strains were named *Apl. kunkeei* S1, *Lpb. plantarum* S2, and *Lcb. pentosus* S3. The genomic functional annotation of these isolated strains was performed using the KEGG, GO, NR, CAZy, and CARD databases. Additionally, the Swiss-Prot and Pfam databases were employed to enhance the understanding of the biological roles of the genes [74,75]. The genomic analysis revealed that the bacteria obtained from the bee bread were closely related to the standard strains of *Apl. kunkeei*, *Lpb. plantarum*, and *Lcb. pentosus*, with an average nucleotide identity (ANI) of 99.95%. Subsequently, the complete genome sequence was annotated to identify genes associated with carbohydrate metabolism, specifically those that enable LAB sp. to adapt to the human gastrointestinal tract (GIT). These genes play a crucial role in the breakdown and absorption of complex carbohydrates that human enzymes cannot digest, serving as significant markers of bacterial adaptation to the gastrointestinal environment.

Our analysis of the genomes of LAB sp. S1, S2, and S3 revealed several important insights. None of the strains showed significant evidence of carrying major virulence factors commonly associated with pathogenicity, such as toxin production, invasion-related genes, or adhesion factors typically found in harmful pathogens. The absence of these factors supports the general safety of the strains for probiotic use. The genome of S1 contained genes related to antibiotic resistance, including the QacG gene associated with resistance to quaternary ammonium compounds. These genes are part of the Small Multidrug Resistance (SMR) family and are primarily involved in resistance to disinfectants and antiseptics. Although these resistance genes may not directly contribute to pathogenicity, their presence highlights the need to assess the potential for horizontal gene transfer and their relevance to clinical safety. S1 was found to harbor genes encoding cytochrome P450 enzymes, which play a role in metabolizing both endogenous and exogenous compounds. These enzymes are generally not associated with virulence, but may contribute to the strain’s ability to metabolize a range of substrates, including potential xenobiotics, which could be of interest for probiotic functionality. The VFDB comparison confirmed that our strains did not carry the major pathogenicity islands (PAIs) or known virulence factor gene clusters (e.g., enterotoxins, cytotoxins, or virulence-associated pili) typically found in harmful bacteria such as *Escherichia coli*, *Salmonella*, or *Streptococcus* species. In case of our strains, the genome of S1 contains a large superfamily of heme-containing monooxygenases called cytochrome P450. In contrast, no such genes were discovered in the genomes of S2 and S3. Similarly, the genome of S1 contains the quaternary ammonium compound-resistance gene QacG, which is responsible for resistance to disinfectants and antiseptics (drug class), and is a member of the AMR Gene family, which is a small multidrug resistance (SMR) antibiotic efflux pump (Table S7). QacC and its related proteins, QacG, QacH, and QacJ, belong to the Small Multi-Resistance Protein (SMR) family. These proteins are typically around 100 amino acids long and feature two transmembrane domains. They are considered to form dimers within the membrane, creating pores that facilitate the expulsion of substrates from the cell. In the *Staphylococcus* species, four classes of SMR-type qac gene families have been identified: qacC, qacG, qacJ, and qacH [9,76]. Within this class, these genes are highly conserved. Nevertheless, qacC genes exhibit a high level of conservation, despite being present in diverse plasmid backgrounds. The disparity in sequence identity among these plasmids, in contrast to the consistent conservation of qacC, suggests the recent dissemination of this gene. The genes responsible for nutrient acquisition and utilization can serve as indicators of the adaptability of a strain to various environments. The TCDB annotation of target protein sequences revealed distinct membrane transport profiles among our strains. Strains S1 and S2 exhibited similar transport protein families, indicating comparable transport capabilities, while strain S3 displayed a different set of transport proteins. This suggests that the variations in membrane transport functions may contribute to the unique phenotypic characteristics observed in each strain.

Before considering probiotics as functional additives, it is essential to conduct comprehensive analysis of the entire genomic sequence of a specific microorganism to ascertain its genotype and phenotype. Identifying the genus and species of a probiotic strain is crucial, as strain-specific probiotics produce distinct probiotic effects. Transferrable genes that confer antibiotic resistance are absent in potential probiotic bacteria, making them unsuitable for assessing the safety of human health [77,78]. A crucial requirement for probiotics is the susceptibility of LAB strains to antibiotics, and there is ongoing debate about whether strains considered safe should exhibit antibiotic sensitivity or resistance. Our safety assessments revealed that the isolated LAB strains were resistant to kanamycin and streptomycin, but they were susceptible to erythromycin, vancomycin, chloramphenicol, and ampicillin. LAB are generally known to be sensitive to chloramphenicol, although some species like *L. acidophilus, Ljl. johnsonii*, and *Lml. reuteri* have shown resistance to this antibiotic [70,79]. Hemolytic activity tests showed that strain S3 exhibited α-hemolysis, while strains S1 and S2 showed γ-hemolysis, suggesting a non-hazardous profile for the latter two strains. Strains S1 and S2 also exhibited the highest survival rates in the presence of 0.5% and 1% bile salts, indicating their potential for use as probiotics. These findings are significant because the acidic environment of the stomach (pH 2.5) and bile salts are key challenges for probiotics, and the ability to withstand these conditions is essential for effective colonization of the gastrointestinal tract. The ideal pH for probiotic survival is around pH 3, as food often neutralizes stomach acid [78]. All three strains demonstrated tolerance to acidic conditions and bile salts, further supporting their potential as probiotics. Moreover, this study provides valuable insights into the hemolytic activity of the target strains when used as probiotics, which has not been widely explored. Probiotic microorganisms thrive during fermentation and in the gastrointestinal tract, where they release antimicrobial compounds such as organic acids, hydrogen peroxide, diacetyl, ethanol, phenols, and bacteriocins. These compounds help eliminate harmful bacteria through a process known as competitive exclusion [80,81]. Given the potential of these LAB strains, further studies are necessary to confirm their safety in vivo and to fully evaluate their probiotic benefits. These findings could have significant implications for the food industry, providing a foundation for developing safer and more effective probiotics [67].

Research indicates that the use of probiotics is supported by the development of specific antibiotic resistance. Probiotics can be given alongside antibiotic treatment to help restore the balance of gut microbes. However, it is crucial that probiotics are safe for human consumption and do not contain genes that promote antibiotic resistance [80]. Our research revealed that lactic acid bacteria (LABs) obtained from stevia fermentation demonstrated both antagonistic activity and probiotic potential [82]. All three strains (S1, S2, and S3) possess the necessary aggregation, adhesion, and hydrophobicity to adhere to the gastrointestinal tract and colonize the large intestine, where they can reduce the presence of pathogenic bacteria. The ability of the LAB strains to inhibit coaggregation with foodborne pathogenic bacteria is attributed to their genetic characteristics. However, the strain’s potential health-promoting properties are likely influenced by its genetic makeup and interactions within its biological environment. Thus, complete sequencing and annotation of the genome will improve our understanding of the strain’s functionality, its adaptation to the human gastrointestinal tract (GIT), its probiotic effects, and its interactions within the host [45]. These microorganisms can alter GI ecology upon administration. Probiotics can impact both mucosal and systemic immune responses. They limit the growth and attachment of harmful organisms, aiding the gut-associated lymphoid tissue [11,39,83]. Nitrites have been found to have carcinogenic potential. Therefore, reducing the levels of nitrites in food is essential for ensuring food safety. However, nitrites are commonly used as colorants and to inhibit the growth of *Clostridium botulinum* in the meat industry. Therefore, current food safety research is focused on finding ways to decrease nitrite levels in food. One possible approach is the inoculation of LAB during the fermentation of certain meals, as this has been shown to effectively prevent the accumulation of high levels of nitrites [24,28]. The three identified strains used in this study were cultured on an MRS medium containing 120 g·mL^−1^ of nitrite, and all the LAB strains showed the greatest ability to degrade nitrite.

The fermentation of LAB strains S1, S2, and S3 with stevia and steviosides led to significant growth, changes in pH, and alterations in amino acid profiles. Strains S2 and S3 showed the strongest growth, especially at lower concentrations of stevia powder and stevioside. Notably, strain S3 had a marked effect on pH at higher stevioside concentrations. In terms of amino acids, LAB S1 increased valine and methionine by 1.67 and 3.15 times, respectively, while essential amino acids generally decreased, except for threonine. Strain S2 had the highest increase in essential amino acids, reaching 293.41 mg/L (30% of total free amino acids), with increases in valine, methionine, isoleucine, leucine, and lysine ranging from 1.95 to 3.47 times. Strain S3 exhibited a 1.26-fold increase in isoleucine during stevia fermentation, while during stevioside fermentation, valine, and lysine levels increased by 2.63-fold and 1.72-fold, respectively. These changes in amino acid composition during fermentation enhance the nutritional value and flavor potential of the final product.

During fermentation, it is essential to utilize lactic acid bacteria (LAB) that can efficiently produce acid, as they play a critical role in rapidly lowering the pH of the fermentation broth, inhibiting the growth of unwanted microbes and enhancing overall fermentation performance. The surface components of bacterial cells are involved in the ability of intact cells to scavenge free radicals. Our study reveals that the isolates exhibit increased scavenging activity, consistent with previous research. The generation of free radicals has been associated with the development and advancement of diabetes [54]. Hydroxyl and similar radicals, which cause oxidative harm to biomolecules, are considered the most dangerous reactive oxygen species [84]. By examining the functions of α-glucosidase and α-amylase, we can reasonably expect a reduction in glucose synthesis and a gradual decrease in the absorption of elevated blood sugar following meals in the small intestine. High concentrations of probiotic bacterial cells are necessary to ensure positive health impacts, making it vital to promote rapid growth and maximize acidification capacity. This study focused on LAB strains S1, S2, and S3, which serve as both probiotics and technological starter cultures. We evaluated their potential to inhibit mycotoxigenic fungi and the enzymes α-glucosidase and α-amylase, crucial for carbohydrate breakdown. The isolates exhibited increased scavenging activity, consistent with previous research linking free radical generation to diabetes development. Hydroxyl radicals, known for causing oxidative damage to biomolecules, are particularly harmful reactive oxygen species. Antioxidants neutralize these radicals through electron or hydrogen atom donation, as demonstrated by DPPH and ABTS assays. Our findings indicate that intact cells from LAB strains S1, S2, and S3 possess significantly higher ABTS radical scavenging abilities compared to cell-free extracts and supernatants. Previous studies have identified various antioxidant molecules in LAB strains, including exopolysaccharides, manganese ions, bioactive compounds, antioxidant enzymes, NADH, and NADPH [84,85]. Previous studies have identified various antioxidant molecules in lactic acid bacteria (LAB) strains, including exopolysaccharides, manganese ions, bioactive compounds, antioxidant enzymes, NADH, and NADPH [86]. Our results align with these findings, demonstrating that LAB strains S1, S2, and S3 also exhibit significant antioxidant activity. For instance, while some studies reported that LAB can scavenge reactive oxygen species (ROS) and enhance antioxidant enzyme levels through various mechanisms, our strains showed a comparable capacity for radical scavenging, particularly against ABTS radicals. Additionally, previous research highlighted the strain-specific nature of antioxidant properties, which is consistent with our observation that S2 exhibited the highest increase in essential amino acids and antioxidant activity among the tested strains. Overall, our findings support the notion that LAB possess substantial antioxidant potential, reinforcing their role as functional probiotics in food applications.

Diabetes has rapidly become a global epidemic, with the number of affected adults projected to rise from 194 million in 2010 to approximately 800 million by 2024 [57,87]. This increase poses significant threats to healthcare systems and economies worldwide, particularly in countries like the United States, China, and India. Individuals with diabetes struggle to manage blood glucose levels, leading to both short- and long-term health complications [57,88]. Targeting carbohydrate-hydrolyzing enzymes, specifically α-glucosidase and α-amylase, is a promising therapeutic strategy for managing postprandial blood glucose levels. Inhibiting these enzymes can reduce glucose absorption and subsequent insulin production. While synthetic inhibitors such as acarbose are available, natural alternatives are being explored for their efficacy and lower side effects. Previous studies have reported various degrees of enzyme inhibition by different probiotic strains; for example, strains CCFM147 and CCFM240 showed α-glucosidase inhibition rates of 27.9% and 32.9%, respectively [19,89]. In contrast, our research found that S1 exhibited a higher inhibition potential of 59.45% for α-glucosidase and 63.35% for α-amylase, outperforming many previously studied strains. Additionally, LAB species like *Lpb. plantarum* are recognized for their ability to regulate gut flora and produce beneficial metabolites without posing health risks. LAB strains exhibit antifungal activity primarily through the production of organic acids and other antimicrobial compounds. Key compounds include lactic acid, acetic acid, and phenyllactic acid, which inhibit fungal growth by lowering the pH and creating an unfavorable environment for fungi [19]. The selected fungal strains *A. flavus*, *P. chrysogenum*, *C. albicans*, and *F. oxysporum* were chosen due to their prevalence in food spoilage and pathogenicity, making them important for assessing the broad-spectrum antifungal activity of lactic acid bacteria (LAB). In addition to organic acids, LAB may produce specific antifungal peptides and secondary metabolites that enhance their inhibitory effects against pathogenic fungi. To effectively incorporate LAB as antifungal agents, further studies are needed to evaluate their effectiveness and explore various application methods, particularly in the contexts of antifungal, antidiabetic, anticancer properties, and bio-preservation.

In a nutshell, our strains S1 (*Apl. kunkeei*), S2 (*Lpb. plantarum*), and S3 (*Lcb. pentosus*) demonstrate greater antioxidant and antifungal activities compared to several other probiotic strains. For instance, while *L. acidophilus* and *Bifidobacterium longum* show notable antioxidant properties, our strains exhibit higher scavenging capabilities against ABTS radicals, indicating enhanced protective effects against oxidative stress [90,91]. Additionally, compared to *Lpb. plantarum Y44*, which has been recognized for its antioxidant activities [92,93], our strains show a broader spectrum of inhibition against mycotoxigenic fungi and enzymes like α-glucosidase and α-amylase [43,94]. Furthermore, unlike some strains that primarily function as probiotics, our strains also serve effectively as technological starter cultures, enhancing their utility in food applications [95]. Overall, the robust antioxidant and antimicrobial properties of strains S1, S2, and S3 position them as highly effective candidates for health-promoting probiotic applications.

Previous studies have demonstrated that *Lactobacillus rhamnosus GG* is known for its high survivability in acidic environments and its ability to inhibit pathogenic bacteria, characteristics that are also observed in *Apl. kunkeei*. Similarly, both *Lpb. plantarum* and *Lcb. pentosus* exhibit strong acid and bile resistance, making them suitable candidates for gut health applications. By contextualizing our findings within the framework of existing research, we highlight the potential of our isolated strains for use in functional foods, particularly in relation to stevia, an area that remains largely unexplored. This comparison highlights the relevance of our work and suggests avenues for future research. However, further investigation into the bacterial composition of bee bread and its fermentation with stevia sweeteners is essential, as it offers valuable insights for developing probiotic products derived from bee bread and its secondary processing, potentially increasing economic value and applications. Fermented stevia, particularly when processed by *Lpb. plantarum*, *Lcb. pentosus*, and *Apl. kunkeei*, has shown promise in exhibiting resistance against microorganisms and cancer cell lines in vitro. Specifically, steviol, a compound derived from stevia, has demonstrated anti-proliferative effects on various human gastrointestinal cancer cell lines and has been evaluated for its anticancer activity against different tumors [3,4]. However, the potential negative interactions between stevia and LAB strains primarily involve the inhibitory effects of stevia glycosides, such as stevioside and rebaudioside A, on the growth of certain LAB species. Research indicates that these glycosides can impair the growth of *Lactobacillus reuteri* strains, with the inhibitory effect being strain-specific and dependent on the concentration of stevia used. For instance, studies have shown that both stevioside and rebaudioside A hinder lactic and acetic acid production in these strains, which are critical for their probiotic function and overall health benefits [52].

### Limitations

The collection of bee bread samples from a single geographical region (Suzhou, China) may limit the generalizability of our findings. Variations in environmental conditions, floral diversity, and microbial communities across different regions can significantly influence the characteristics and probiotic potential of lactic acid bacteria. Future studies should aim to include samples from multiple geographical locations to enhance the applicability of the results. Probiotics have been widely studied in recent years, with research conducted using both in vitro and in vivo methods, as well as clinical applications. However, there is a need to further explore the genetic characteristics of probiotics. In addition to in vitro studies, extensive evaluations within the human body and the use of laboratory models are important for enhancing our understanding. Researchers are currently seeking new strategies to combat infectious diseases caused by antibiotic misuse and the emergence of resistant microbes. Therefore, identifying lactic acid bacteria (LAB) is a crucial initial step toward the industrial production of these bacteria, which can help address infectious diseases in humans and animals. The use of inexpensive raw materials in the fermentative production of lactic acid adds to its appeal. Raw materials such as molasses, sucrose, maltose, and glucose can serve as carbon sources for the fermentation process. The present study found that *Apl. kunkeei*, *Lpb. plantarum*, and *Lcb. pentosus* achieved their maximum growth and lactic acid production at high glucose concentrations. An intriguing area of study involves isolating LAB strains with probiotic potential and exploring the effects of stevia and steviosides. *LAB* species are well known for their probiotic properties, which provide health benefits to the host when consumed. The isolation of LAB with probiotic potential, along with the study of stevia leaves and sweeteners, represents a promising area of research. This work has the potential to lead to the development of innovative probiotic products that offer enhanced health benefits. It is essential to investigate how stevia glycosides influence the metabolism of probiotic bacteria to clarify the observed effects. Given the strain-specific enhancements or inhibitory effects of stevia glycosides, it is advisable to test their impacts on each strain individually before incorporating them into functional foods.

## 5. Conclusions

In conclusion, this study investigated three novel LAB strains (*Apl. kunkeei*, *Lpb. plantarum*, and *Lcb. Pentosus*) that were isolated from bee bread in Suzhou, China. Whole genome sequencing of these strains revealed their significant potential for applications in the food and feed industries. The isolation of these probiotic bacteria, recognized for their health benefits, offers valuable insights for advancing innovative products in both the food and pharmaceutical sectors. Our findings suggest that these lactic acid bacteria (LAB) isolates are promising candidates for use as probiotics in humans and animals, as well as effective starter cultures for food production. Future research should focus on the unique LAB present in bee bread, examining key taxa and their ecological roles. Additionally, a deeper exploration of the functional genes associated with various metabolic pathways in *Lcb. pentosus* and *Lpb. plantarum* is warranted to enhance our understanding of their capabilities. The observed variations in growth patterns during fermentation with stevia and steviosides can be attributed to the distinct characteristics of each strain. However, it is important to acknowledge that while our findings are promising, further in vivo studies are necessary to validate the efficacy and safety of these LAB strains in real-world applications. Conducting such studies will be crucial to fully understand the effects of stevia glycosides on commonly used probiotic strains in both in vitro and in vivo environments. This investigation lays the groundwork for developing or expanding novel fermented stevia-based functional products and their derivatives within the food and pharmaceutical industries.

## Figures and Tables

**Table 1:** Component volume of one reaction.

PCR product	2.5 µL
2×KAPA HiFi Ready Mix	12.5 µL
Forward primer (25 µM)	0.25 µL
Reverse primer (25 µM)	0.25 µL
PCR-grade water	9.5 µL
Total volume	25 µL

**Table 2:** The characteristics and carbohydrate fermentation patterns of three LAB strains.

Characteristic	Isolated Strains		
	S1	S2	S3
Growth in MRS at:			
15 °C	−	w	−
45 °C	−	−	−
The Optimum growth temperature (°C)	30	30	30
pH 4.0	++	++	++
pH 3.5	+	+	+
pH 3.0	−	+	w
pH 2.5	−	−	w
pH 2.0	−	−	−
6%	+	w	w
8%	w	−	w
10%	−	−	−
Others:			
Motility	−	−	−
Gram-stain	+	+	+
Catalase assay	−	−	−
H_2_S production test	−	−	−
Gelatin liquefaction test	−	−	−
Indole production test	−	−	−
Starch hydrolysis test	+	+	+
Acid production from:			
L-arabinose	+	+	+
D-cellulose-disaccharide	+	+	+
D-galactose	+	+	+
D-lactose	++	++	++
D-maltose	+	++	++
D-mannose	+	++	+
D-mannitol	+	+	+
D-triose	−	+	−
L-rhamnose	−	−	−
D-ribose	+	+	+
D-sucrose	+	+	+
D-xylose	−	−	−
L-xylose	−	−	−
D-fructose	+	+	+
D-sorbitol	+	+	+
L-sorbose	−	−	−
inositol	−	−	−
glycerol	+	+	+

++: strong positive; +: positive; w: weak positive; −: negative.

**Table 3:** Inhibition ability of LAB isolates against three indicator bacteria.

Strain	Zone of Inhibition Size (mm)		
	*Escherichia coli*	*Salmonella*	*Staphylococcus aureus*
S1	12.67 ± 1.04	−	15.08 ± 1.01
S2	15.77 ± 0.84	9.55 ± 1.12	11.04 ± 0.98
S3	13.35 ± 1.04	11.02 ± 0.54	10.68 ± 0.22

**Table 4:** Drug sensitivity and auto aggregation.

Strain	Drug Sensitivity							Auto-Aggregation (%)
	GEN	S	KAN	ERY	C	AMP	VA	
S1	S	S	R	R	S	S	S	17.43 ± 4.16
S2	S	S	R	R	S	S	S	28.14 ± 3.85
S3	S	S	R	R	S	S	S	32.14 ± 3.48

**Table 5:** Inhibitory activity of the isolates against α-glucosidase and α-amylase.

Isolates	α-Glucosidase Inhibition * (CS)	(IC)	α-Amylase Inhibition * (CS)	(IC)
**S1**	49.35 ± 1.97 a	21.50 ± 2.23 a	59.35 ± 2.34 b	32.25 ± 1.97 c
**S2**	52.42 ± 2.47 b	25.57 ± 2.45 b	58.21 ± 3.24 b	34.24 ± 2.47 d
**S3**	59.45 ± 1.84 c	26.35 ± 2.06 c	63.35 ± 3.24 d	36.12 ± 1.84 d

* The data is expressed as mean ± SD. According to Duncan’s multiple range test, the means within the same column that are represented by distinct letters (a–d) are significantly different (*p* ≤ 0.05).

## Data Availability

The data presented in this study are openly available in (https://www.ncbi.nlm.nih.gov). The 16S rRNA gene sequences were deposited in GenBank, with accession numbers CP128865, CP134769, and CP134770 for *Apilactobacillus kunkeei* (S1), *Lactiplanti-bacillus plantarum* strain (S2), and *Lacticaseibacillus pentosus* (S3), respectively.

## Supplementary Materials

The following supporting information can be downloaded at: https://www.mdpi.com/article/10.3390/microorganisms13020216/s1, Figure S1 (a,b,c). The complete genomes of three strains, S1, S2, and S3, consist of circular chromosomes with the following sizes: 1,534,266 bp, 2,839,103 bp, and 3,246,259 bp, respectively. Figure S2 (a, b, c). Predicting the distribution of matching species in the database. Figure S3. The GO classification system of *Apl. kunkeei*, *Lpb. Plantarum*, and *Lcb. pentosus, respectively.* Figure S4. (a) Hemolytic activity of three strains. (a) Isolates with a green halo were designated as α-hemolytic, (b) isolates presenting no halo were classified as γ-hemolytic. Our study demonstrates *Apl. kunkeei* and *Lpb. plantarum* shows γ–hemolysis. While *Lcb. pentosus* S3 show α-hemolytic. Table S1. Annotation state information of *Apl. kunkeei*. Table S2. Annotation state information of *Lpb. plantarum*. Table S3. Annotation state information of *Lcb. pentosus*. Table S4. Functional gene annotation of *Apb. kunkeei*. Table S5. CARD annotation table of S1. Table S6 (a) PHI annotation table of S1. Table S6 (b) PHI annotation table of S2. Table S6 (c) PHI annotation table. Table S7 (a) VFDB annotation table of S1. Table S7 (b). VFDB annotation table of S2. Table S7 (c) VFDB annotation table of S3. Table S9 (a). CYPED annotation table of S1. Table S8 (a). Cazy database results table of S1. Table S8 (b). Cazy database results table of S2 (P). Table S8 (c). Cazy database results table of S3. Table S9 (b). CYPED annotation table of S2. Table S9 (c). CYPED annotation table of S3. Table S10. Tolerance to artificial simulated gastrointestinal conditions and cell surface hydrophobicity of *Lactobacillus* spp. Table S11. Ability of the isolated strains to degrade nitrite. Table S12 (a). Biomass of Stevia fermented by LAB. Table S12 (b). Biomass of Steviosides fermented by LAB. Table S13 (a). Concentration of free amino acids in Stevia fermentation broth at the beginning and end of fermentation. Table S13 (b). Fermentation broth of each strain with steviosides.
