# Supplementary material for: Screening and Comparative Genomics of Probiotic Lactic Acid Bacteria from Bee Bread of Apis Cerana: Influence of Stevia and Stevioside on Bacterial Cell Growth and the Potential of Fermented Stevia as an Antidiabetic, Antioxidant, and Antifungal Agent
Source: Microorganisms. 2025 Jan 21;13(2):216. doi: 10.3390/microorganisms13020216 (PMC11857352; doi:10.3390/microorganisms13020216)
Supplement: Supplementary file 1 [file microorganisms-13-00216-s001.zip › microorganisms-3444750-supplementary.pdf]

Supplementary material:

Figure S1 (a,b,c). The complete genomes of three strains, S1, S2, and S3, consist of circular chromosomes with the following sizes: 1,534,266 bp, 2,839,103 bp, and 3,246,259 bp, respectively. Figure S2 (a, b, c). Predicting the distribution of matching species in the database. Figure S3. The GO classification system of *Apl. kunkeei*, *Lpb. Plantarum*, and *Lcb. pentosus*, respectively. Figure S4. (a) Hemolytic activity of three strains. (a) Isolates with a green halo were designated as α-hemolytic, (b) isolates presenting no halo were classified as γ-hemolytic. Our study demonstrates *Apl. kunkeei* and *Lpb. plantarum* shows γ–hemolysis.

While *Lcb. pentosus* S3 show α-hemolytic. Table S1. Annotation state information of *Apl. kunkeei*. Table S2. Annotation state information of *Lpb. plantarum*. Table S3. Annotation state information of *Lcb. pentosus*. Table S4. Functional gene annotation of *Apb. kunkeei*. Table S5. CARD annotation table of S1. Table S6 (a) PHI annotation table of S1. Table S6 (b) PHI annotation table of S2. Table S6 (c) PHI annotation table. Table S7 (a) VFDB annotation table of S1. Table S7 (b). VFDB annotation table of S2. Table S7 (c) VFDB annotation table of S3.

Table S9 (a). CYPED annotation table of S1. Table S8 (a). Cazy database results table of S1. Table S8 (b). Cazy database results table of S2 (P). Table S8 (c). Cazy database results table of S3. Table S9 (b). CYPED annotation table of S2. Table S9 (c). CYPED annotation table of S3. Table S10. Tolerance to artificial simulated gastrointestinal conditions and cell surface hydrophobicity of *Lactobacillus* spp. Table S11. Ability of the isolated strains to degrade nitrite. Table S12 (a). Biomass of Stevia fermented by LAB. Table S12 (b). Biomass of Steviosides fermented by LAB. Table S13 (a). Concentration of free amino acids in Stevia fermentation broth at the beginning and end of fermentation. Table S13 (b). Fermentation broth of each strain with steviosides.

Table S1. Annotation state information of *Apilactobacillus kunkeei* S1

	Type	Number	Total_length	Average_length	Percentage_of_genome(%)
**1**	16S rRNA	5	7,845	1,569	0.51
**2**	23S rRNA	5	14,575	2,915	0.95
**3**	5S rRNA	5	555	111	0.04
**4**	CDS	1,376	1,371,438	997	89.04
**5**	CRISPR	1	1,422	1,422	0.09
**6**	tRNA	65	5,017	77	0.33
**7**	tmRNA	1	368	368	0.02

Table S2. Annotation state information of *Lactiplantibacillus plantarum* SYBC.SM2

	Type	Number	Total_length	Average_length	Percentage_of_genome(%)
**1**	16S rRNA	6	8,558	1,426	0.29
**2**	23S rRNA	5	14,585	2,917	0.5
**3**	5S rRNA	6	672	112	0.02
**4**	CDS	2,795	2,444,565	875	83.19
**5**	tRNA	85	6,500	76	0.22
**6**	tmRNA	1	369	369	0.01

Table S3. Annotation state information of *Lacticaseibacillus pentosus SYBC.SM3*

	Type	Number	Total_length	Average_length	Percentage_of_genome(%)
**1**	16S rRNA	5	7,815	1,563	0.24
**2**	23S rRNA	5	14,585	2,917	0.45
**3**	5S rRNA	6	672	112	0.02
**4**	CDS	3,050	2,747,193	901	84.33
**5**	tRNA	72	5,551	77	0.17
**6**	tmRNA	1	369	369	0.01

Table.S4. Functional gene annotation of *Apl. kunkeei*

Type	Number of genes S1	S2	S3
NR	1,368	2,776	3,040
KEGG	939	1,594	1,676
GO	346	450	455
Swiss Prot	978	1,824	1,817
TrEMBL	1,370	2,779	3,037
COG	1,149	2,301	2,399
PFAM	1,216	2,442	2,578

Table S5. CARD annotation table of S1

	**ORF_ID**	**Best_Hit_ARO**	**Best_Identities**	**ARO**	**Drug Class**	**Resistance Mechanism**	**AMR Gene Family**
1	IALLNKNO_01251 Quaternary ammonium compound-resistance protein QacC	qacG	52.38	3007015	disinfecting agents and antiseptics	antibiotic efflux	small multidrug resistance (SMR) antibiotic efflux pump

Table S8 (a). Cazy database results table of S1				
	**Gene ID**	**HMMER**	**Hotpep**	**DIAMOND**
1	IALLNKNO_00058	GT2_Glyco_trans_2_3(136-348)	-	GT2
2	IALLNKNO_00070	GH65(290-662)	GH65(3)	GH65
3	IALLNKNO_00097	GT2_Glycos_transf_2(9-170)	GT2(245)	GT2
4	IALLNKNO_00098	GT83(19-236)	-	GT0
5	IALLNKNO_00102	GH25(40-204)	GH25(42)	GH25
6	IALLNKNO_00134	GH32(40-344)	GH32(2)	GH32
7	IALLNKNO_00190	GH73(71-216)	GH73(111)	GH73
8	IALLNKNO_00219	GT4(327-476)	-	GT4
9	IALLNKNO_00220	GT4(332-490)	-	GT4
10	IALLNKNO_00260	CE4(126-253)	-	CE4

Table S8 (b). Cazy database results table of S2 (P)				
	**Gene ID**	**HMMER**	**Hotpep**	**DIAMOND**
1	HACOMAID_00019	CBM48(27-106)+GH13_9(174-473)	GH13(1)+CBM48(2)	CBM48+GH13_9
2	HACOMAID_00022	GT5(3-476)	GT5(3)	GT5
3	HACOMAID_00023	GT35(93-784)	GT35(1)	GT35
4	HACOMAID_00024	GH13_39(188-496)	GH13(24)+GH77(17)+CBM20(8)+CBM34(3)+CBM48(57)	GH13_39
5	HACOMAID_00028	GH65(427-836)	GH65(4)	GH65
6	HACOMAID_00147	GH36(14-593)	GH36(47)	GH36
7	HACOMAID_00152	GH32(37-339)	GH32(2)	GH32
8	HACOMAID_00154	GH13_31(30-378)	GH13(3)	GH13_31
9	HACOMAID_00157	GH13_31(28-379)	GH13(16)	GH13_31
10	HACOMAID_00216	GH13_29(24-370)	GH13(44)	GH13_29

Table S8 (c). Cazy database results table of S3

	Gene ID	HMMER	Hotpep	DIAMOND
1	NDCMEIBK_00018	CBM48(27-106)+GH13_9(174-473)	GH13(1)+CBM48(2)	CBM48+GH13_9
2	NDCMEIBK_00021	GT5(3-476)	GT5(3)	GT5
3	NDCMEIBK_00022	GT35(93-792)	GT35(1)	GT35
4	NDCMEIBK_00023	GH13_39(188-496)	GH13(24)+GH77(17)+CBM20(8)+CBM34(3)+CBM48(57)	GH13_39
5	NDCMEIBK_00027	GH65(427-836)	GH65(4)	GH65
6	NDCMEIBK_00158	GH13_31(28-384)	GH13(39)	GH13
7	NDCMEIBK_00163	GH13(25-314)	-	GH13
8	NDCMEIBK_00165	GH65(322-693)	GH65(1)	GH65
9	NDCMEIBK_00167	GH85(109-451)+CBM32(813-938)	GH85(1)+CBM32(4)	CBM32+GH85
10	NDCMEIBK_00170	GH36(12-700)	GH36(47)	GH36

Table S6 (a) PHI annotation table of S1

	s-seqid	PHI_info of S1
1	A0A0H3PAK7	PHI:10727 PepP_(CJJ81176_0681) 197 Campylobacter_jejuni reduced_virulence
2	D2TI55	PHI:9334 Map 67825 Citrobacter_rodentium effector_(plant_avirulence_determinant)
3	Q75WC5	PHI:824__PHI:6838 GyrA 337 Burkholderia_glumae chemistry_target: _sensitivity_to_chemical__chemistry_tar
4	Q75WC1	PHI:849 GyrA 337 Burkholderia_glumae chemistry_target: _resistance_to_chemical__sensitivity_to_chemical
5	Q75WC4	PHI:852 GyrA 337 Burkholderia_glumae chemistry_target: _resistance_to_chemical__sensitivity_to_chemical
6	Q75WC2	PHI:850 GyrA 337 Burkholderia_glumae chemistry_target: _resistance_to_chemical_sensitivity_to_chemical
7	Q75WC3	PHI:851 GyrA 337 Burkholderia_glumae chemistry_target: _resistance_to_chemical_sensitivity_to_chemical
8	Q04HX1	PHI:4222 Pde1 1313 Streptococcus_pneumoniae reduced_virulence
9	Q2YKG0	PHI:9130 Bab2_0700 235 Brucella_abortus reduced_virulence
10	A0A0H2ZNJ1	PHI:9397 AliB_(SPD_1357) 1313 Streptococcus_pneumoniae reduced_virulence

Table S6 (b) PHI annotation table of S2

	sseqid	PHI_info
1	Q75WC5	GyrA 337 Burkholderia_ glumae chemistry_target:_sensitivity to chemical, l reduced_virulence__loss of pathog
2	Q75WC1	GyrA 337 Burkholderia_glumae chemistry_target: _resistance_to_chemical, sensitivity to chemical
3	Q75WC4	GyrA 337 Burkholderia_glumae chemistry_target: _resistance_to_chemical, sensitivity to chemical
4	Q75WC2	GyrA 337 Burkholderia_glumae chemistry_target: _resistance_to_chemical-sensitivity to chemical
5	Q75WC3	GyrA 337 Burkholderia_glumae chemistry_target: _resistance_to_chemical_sensitivity to chemical
6	Q5H2X8	SSBX 347 Xanthomonas_oryzae reduced_virulence
7	Q04HX1	Pde1 1313 Streptococcus_pneumoniae reduced_virulence
8	A5ICA4	ProA 446 Legionella_pneumophila reduced_virulence
9	Q2YKG0	Bab2_0700 235 Brucella_abortus reduced_virulence
10	A0A0H2Z NJ1	AliB_(SPD_1357) 1313 Streptococcus_pneumoniae reduced_virulence

Table S6 (c) PHI annotation table

	sseqid	PHI_info
1	Q75WC5	PHI:824__PHI:6838 GyrA 337 Burkholderia_glumae chemistry_target:_sensitivity_to_chemical__chemistry_target:_resistance_to_chemical__reduced_virulence_loss
2	Q75WC1	PHI:849 GyrA 337 Burkholderia_glumae chemistry_target:_resistance_to_chemical___sensitivity_to_chemical
3	Q75WC4	PHI:852 GyrA 337 Burkholderia_glumae chemistry_target:_resistance_to_chemical__sensitivity_to_chemical
4	Q75WC2	PHI:850 GyrA 337 Burkholderia_glumae chemistry_target:_resistance_to_chemical__sensitivity_to_chemical
5	Q75WC3	PHI:851 GyrA 337 Burkholderia_glumae chemistry_target:_resistance_to_chemical__sensitivity_to_chemical
6	Q5H2X8	PHI:2737 SSBX 347 Xanthomonas_oryzae reduced_virulence
7	Q04HX1	PHI:4222 Pde1 1313 Streptococcus_pneumoniae reduced_virulence
8	A5ICA4	PHI:11263 ProA 446 Legionella_pneumophila reduced_virulence
9	Q2YKG0	PHI:9130 Bab2_0700 235 Brucella_abortus reduced_virulence
10	A0A0H2Z NJ1	PHI:9397 AliB_(SPD_1357) 1313 Streptococcus_pneumoniae reduced_virulence

Table S9 (a). CYPED annotation table of S1

	qseqid	sseqid	CYPED_info
1	IALLNKNO_00039	sid|14348	pid|10538|hfid|1401|sfid|504|gi|629670177|taxonID|1287681|EutypalataUCREL1
2	IALLNKNO_00187	sid|86444	pid|63948|hfid|719|sfid|94|gb|ABA95747.2|gi|108862162|taxonID|39947|OryzasativaJap
3	IALLNKNO_00187	sid|86443	pid|63947|hfid|719|sfid|94|gb|EEC68823.1|gi|218186396|taxonID|39946|OryzasativaInd
4	IALLNKNO_00259	sid|86444	pid|63948|hfid|719|sfid|94|gb|ABA95747.2|gi|108862162|taxonID|39947|OryzasativaJap
5	IALLNKNO_00259	sid|86443	pid|63947|hfid|719|sfid|94|gb|EEC68823.1|gi|218186396|taxonID|39946|OryzasativaInd
6	IALLNKNO_00724	sid|99190	pid|74873|hfid|381|sfid|51|gb|EGC42569.1|gi|325089259|taxonID|544711|Ajellomycesca
7	IALLNKNO_00724	sid|99191	pid|74873|hfid|381|sfid|51|gb|EER36755.1|gi|240273233|taxonID|544712|Ajellomycesca
8	IALLNKNO_00724	sid|99192	pid|74874|hfid|381|sfid|51|gb|EEH02637.1|gi|225554338|taxonID|447093|Ajellomycesca
9	IALLNKNO_00724	sid|86485	pid|63985|hfid|381|sfid|51|gb|EMD85094.1|gi|451992614|taxonID|701091|Bipolarismay
10	IALLNKNO_00724	sid|86482	pid|63984|hfid|381|sfid|51|gb|EUN26126.1|gi|578488685|taxonID|930091|Bipolarisvicto

Table S9 (b). CYPED annotation table of S2

	Qseqid	Sseqid	CYPED_info
1	HACOMAID_00382	pid|63948|hfid|719|sfid|94|gb|ABA95747.2|gi|108862162|taxonID|39947|OryzasativaJaponicaGroup	
2	HACOMAID_00382	pid|63947|hfid|719|sfid|94|gb|EEC68823.1|gi|218186396|taxonID|39946|OryzasativaIndicaGroup	
3	HACOMAID_00433	pid|10538|hfid|1401|sfid|504|gi|629670177|taxonID|1287681|EutypalataUCREL1	
4	HACOMAID_00439	pid|10538|hfid|1401|sfid|504|gi|629670177|taxonID|1287681|EutypalataUCREL1	
5	HACOMAID_00646	pid|74618|hfid|1168|sfid|177|gi|256032277|gi|256032279|pdb|2WIV(A, B)|pdb|2WIY(A)|taxonID|1827|Rhodococcus|gb|AGU90203.1|gi|537801345|taxonID|1394869|Willia	
6	HACOMAID_00646	pid|74618|hfid|1168|sfid|177|gb|ACO88874.1|gi|226860320|taxonID|614068|Microbacteriumsp.MA	
7	HACOMAID_00646	pid|74618|hfid|1168|sfid|177|gi|504737662|gb|ADK69005. 2|gi|411170352|taxonID|337191|Gordon	
8	HACOMAID_00657	pid|63947|hfid|719|sfid|94|gb|EEC68823.1|gi|218186396|taxonID|39946|OryzasativaIndicaGroup	
9	HACOMAID_00657	pid|63948|hfid|719|sfid|94|gb|ABA95747.2|gi|108862162|taxonID|39947|OryzasativaJaponicaGroup	
10	HACOMAID_00675	pid|10538|hfid|1401|sfid|504|gi|629670177|taxonID|1287681|EutypalataUCREL1	

Table S9 (c). CYPED annotation table of S3

	qseqid	sseqid	CYPED_info
1	NDCMEIBK_00508	sid|86443	pid|63947|hfid|719|sfid|94|gb|EEC68823.1|gi|218186396|taxonID|39946|OryzasativaInd
2	NDCMEIBK_00508	sid|86444	pid|63948|hfid|719|sfid|94|gb|ABA95747.2|gi|108862162|taxonID|39947|OryzasativaJap
3	NDCMEIBK_00778	sid|86444	pid|63948|hfid|719|sfid|94|gb|ABA95747.2|gi|108862162|taxonID|39947|OryzasativaJap
4	NDCMEIBK_00779	sid|86443	pid|63947|hfid|719|sfid|94|gb|EEC68823.1|gi|218186396|taxonID|39946|OryzasativaInd
5	NDCMEIBK_00779	sid|86444	pid|63948|hfid|719|sfid|94|gb|ABA95747.2|gi|108862162|taxonID|39947|OryzasativaJap
6	NDCMEIBK_00888	sid|88361	pid|65577|hfid|1422|sfid|512|gb|EGO05195. 1|gi|336376860|taxonID|936435|Serpulalacr
7	NDCMEIBK_01381	sid|99190	pid|74873|hfid|381|sfid|51|gb|EGC42569.1|gi|325089259|taxonID|544711|Ajellomycesca
8	NDCMEIBK_01381	sid|99191	pid|74873|hfid|381|sfid|51|gb|EER36755.1|gi|240273233|taxonID|544712|Ajellomycesca
9	NDCMEIBK_01381	sid|86482	pid|63984|hfid|381|sfid|51|gb|EUN26126.1|gi|578488685|taxonID|930091|Bipolarisvicto
10	NDCMEIBK_01381	sid|86483	pid|63984|hfid|381|sfid|51|gi|628209341|taxonID|930089|Bipolariszeicola26-R-13

Table S7 (a) VFDB annotation table of S1

qseqid		Sseqid	VFDB_
1	IALLNKNO_00018	VFG006784(gb|WP_003769807)	(oppA) peptide ABC transporter substrate-binding protein [Oligopeptide-binding protein oppA (VF0786) - Nutritional/Metabolic factor (VFC0272)] [Listeria innocua Clip11262]
2	IALLNKNO_00018	VFG006783(gb|WP_003724612)	(oppA) peptide ABC transporter substrate-binding protein [Oligopeptide-binding protein oppA (VF0786) - Nutritional/Metabolic factor (VFC0272)] [Listeria monocytogenes serotype 4b str. F2365]
3	IALLNKNO_00018	VFG032247(gb|WP_014601048)	(oppA) peptide ABC transporter substrate-binding protein

			[Oligopeptide-binding protein oppA (VF0786) - Nutritional/Metabolic factor (VFC0272)] [Listeria monocytogenes 10403S]
4	IALLNKNO_00018	VFG006782(gb|NP_465720)	(oppA) hypothetical protein [Oligopeptide-binding protein oppA (VF0786) - Nutritional/Metabolic factor (VFC0272)] [Listeria monocytogenes EGD-e]
5	IALLNKNO_00018	VFG019366(gb|WP_003729598)	(oppA) peptide ABC transporter substrate-binding protein [Oligopeptide-binding protein oppA (VF0786) - Nutritional/Metabolic factor (VFC0272)] [Listeria monocytogenes HCC23]
6	IALLNKNO_00019	VFG001965(gb|YP_002344820)	(Cj1437c) aminotransferase [Capsule (VF0323) - Immune modulation (VFC0258)] [Campylobacter jejuni subsp. jejuni NCTC 11168]
7	IALLNKNO_00019	VFG047305(gb|WP_003040378)	(wbtQ) aminotransferase class I/II-fold pyridoxal phosphate-dependent enzyme [LPS (VF0542) - Immune modulation (VFC0258)] [Francisella novicida U112]
8	IALLNKNO_00019	VFG047306(gb|WP_014548780)	(wbtQ) DegT/DnrJ/EryC1/StrS family aminotransferase [LPS (VF0542) - Immune modulation (VFC0258)] [Francisella cf. tularensis subsp. novicida 3523]
9	IALLNKNO_00030	VFG016003(gb|WP_011062369)	(pvdN) pyoverdine biosynthesis protein PvdN [Pyoverdine (VF0094) - Nutritional/Metabolic factor (VFC0272)] [Pseudomonas fluorescens Pf-5]
10	IALLNKNO_00032	VFG022782(gb|WP_003910332)	(irtA) Iron-regulated transporter IrtA [ABC transporter (VF0818) - Nutritional/Metabolic factor (VFC0272)] [Mycobacterium africanum GM041182]

Table S7 (b). VFDB annotation table of S2

qseqid		Sseqid	VFDB_
1	HACOMAID_00018	VFG032255(gb|WP_003753364)	(oppA) peptide ABC transporter substrate-binding protein [Oligopeptide-binding protein oppA (VF0786) - Nutritional/Metabolic factor (VFC0272)] [Listeria seeligeri serovar 1/2b str. SLCC3954]
2	HACOMAID_00018	VFG006784(gb|WP_003769807)	(oppA) peptide ABC transporter substrate-binding protein [Oligopeptide-binding protein oppA (VF0786) - Nutritional/Metabolic factor (VFC0272)] [Listeria innocua Clip11262]
3	HACOMAID_00018	VFG006785(gb|WP_011702965)	(oppA) peptide ABC transporter substrate-binding protein [Oligopeptide-binding protein oppA (VF0786) - Nutritional/Metabolic factor (VFC0272)] [Listeria welshimeri serovar 6b str. SLCC5334]
4	HACOMAID_00018	VFG032250(gb|WP_003722319)	(oppA) peptide ABC transporter substrate-binding protein [Oligopeptide-binding protein oppA (VF0786) - Nutritional/Metabolic factor (VFC0272)] [Listeria monocytogenes J0161]
5	HACOMAID_00018	VFG032247(gb|WP_014601048)	(oppA) peptide ABC transporter substrate-binding protein [Oligopeptide-binding protein oppA (VF0786) - Nutritional/Metabolic factor (VFC0272)] [Listeria monocytogenes 10403S]
6	HACOMAID_00020	VFG006052(gb|WP_011226150)	(rfbA) glucose-1-phosphate thymidylyltransferase RfbA [Capsule (VF0144) - Immune modulation (VFC0258)] [Streptococcus thermophilus CNRZ1066]

7	HACOMAID_00020	VFG006041(gb|WP_011681286)	(rfbA) glucose-1-phosphate thymidylyltransferase RfbA [Capsule (VF0144) - Immune modulation (VFC0258)] [Streptococcus thermophilus LMD-9]
8	HACOMAID_00020	VFG005997(gb|WP_002263084)	(rfbA) glucose-1-phosphate thymidylyltransferase RfbA [Capsule (VF0144) - Immune modulation (VFC0258)] [Streptococcus mutans UA159]
9	HACOMAID_00020	VFG037938(gb|WP_000104825)	(rfbA) glucose-1-phosphate thymidylyltransferase RfbA [Capsule (VF0465) - Immune modulation (VFC0258)] [Acinetobacter baumannii TYTH-1]
10	HACOMAID_00020	VFG018664(gb|WP_012000426)	(rfbA) glucose-1-phosphate thymidylyltransferase RfbA [Capsule (VF0144) - Immune modulation (VFC0258)] [Streptococcus gordonii str. Challis substr. CH1]

Table S7 (c) VFDB annotation table of S3

**qseqid**		**sseqid**	**VFDB_**
1	NDCMEIBK_00017	VFG032255(gb|WP_003753364)	(oppA) peptide ABC transporter substrate-binding protein [Oligopeptide-binding protein oppA (VF0786) - Nutritional/Metabolic factor (VFC0272)] [Listeria seeligeri serovar 1/2b str. SLCC3954]
2	NDCMEIBK_00017	VFG006784(gb|WP_003769807)	(oppA) peptide ABC transporter substrate-binding protein [Oligopeptide-binding protein oppA (VF0786) - Nutritional/Metabolic factor (VFC0272)] [Listeria innocua Clip11262]

3	NDCMEIBK_00017	VFG006785(gb|WP_011702965)	(oppA) peptide ABC transporter substrate-binding protein [Oligopeptide-binding protein oppA (VF0786) - Nutritional/Metabolic factor (VFC0272)] [Listeria welshimeri serovar 6b str. SLCC5334]
4	NDCMEIBK_00017	VFG032250(gb|WP_003722319)	(oppA) peptide ABC transporter substrate-binding protein [Oligopeptide-binding protein oppA (VF0786) - Nutritional/Metabolic factor (VFC0272)] [Listeria monocytogenes J0161]
5	NDCMEIBK_00017	VFG032247(gb|WP_014601048)	(oppA) peptide ABC transporter substrate-binding protein [Oligopeptide-binding protein oppA (VF0786) - Nutritional/Metabolic factor (VFC0272)] [Listeria monocytogenes 10403S]
6	NDCMEIBK_00019	VFG006052(gb|WP_011226150)	(rfbA) glucose-1-phosphate thymidylyltransferase RfbA [Capsule (VF0144) - Immune modulation (VFC0258)] [Streptococcus thermophilus CNRZ1066]
7	NDCMEIBK_00019	VFG006041(gb|WP_011681286)	(rfbA) glucose-1-phosphate thymidylyltransferase RfbA [Capsule (VF0144) - Immune modulation (VFC0258)] [Streptococcus thermophilus LMD-9]
8	NDCMEIBK_00019	VFG005997(gb|WP_002263084)	(rfbA) glucose-1-phosphate thymidylyltransferase RfbA [Capsule (VF0144) - Immune modulation (VFC0258)] [Streptococcus mutans UA159]
9	NDCMEIBK_00019	VFG037938(gb|WP_000104825)	(rfbA) glucose-1-phosphate thymidylyltransferase RfbA [Capsule (VF0465) - Immune modulation (VFC0258)] [Acinetobacter baumannii TYTH-1]
10	NDCMEIBK_00019	VFG018664(gb|WP_012000426)	(rfbA) glucose-1-phosphate thymidylyltransferase RfbA [Capsule (VF0144) - Immune modulation (VFC0258)] [Streptococcus gordonii str. Challis substr. CH1]

Table S11. Ability of the isolated strains to degrade nitrite

Strain number	Final nitrite content (μg·mL^{-1})	Degradation rate (%)	Final pH value
S1	0.28	95.78	4.01
S2	0.37	96.70	4.03
S3	0.35	96.71	4.03

Table S10. Tolerance to artificial simulated gastrointestinal conditions and cell surface hydrophobicity of *Lactobacillus* spp.

**Strain**	**Artificial simulated gastric juice**		**Cell surface hydrophobicity %**
	24h survival rate %	48h survival rate %	
**S1**	75± 3.66^{ab}	70± 4.45^{ab}	26.07 ± 1.32%
**S2**	70± 3.20^{ab}	72± 4.15^{ab}	25.07± 1.30%
**S3**	72± 3.35^{ab}	70± 4.28^{ab}	23.25± 1.33%

Data are expressed as the mean ± standard deviation of three measurements. Values with the same superscript letters (a, b) indicate no significant difference at a specified significance level (e.g., $p < 0.05$).

Table S12 (a). Biomass of Stevia fermented by LAB

**Fermentation time（d）**	**Biomass of lactic acid bacteria fermented with Stevia leaf powder（CFU·mL^{-1}）**		
	S1	S2	S3
**0**	6.8×10^{7}	6.2×10^{7}	3.7×10^{7}
**2**	6.0×10^{8}	6.3×10^{8}	1.3×10^{8}

**4**	8×10^{8}	1.8×10^{8}	3.0×10^{7}
**6**	6.2×10^{7}	1.5×10^{7}	1.6×10^{7}
**8**	3.3×10^{7}	4.3×10^{7}	7.0×10^{6}
**10**	3.0×10^{7}	8.9×10^{6}	8.9×10^{6}

Table S12(b). Biomass of Steviosides fermented by LAB

**Fermentation time（d）**	**Biomass of LAB fermented with steviosides（CFU·mL^{-1}）**		
	S1	S2	S3
**0**	5.2×10^{7}	5.8×10^{7}	6.0×10^{7}
**2**	6.3×10^{8}	6.7×10^{8}	4.3×10^{8}
**4**	1.8×10^{8}	4.0×10^{8}	3.8×10^{8}
**6**	5.1×10^{7}	1.0×10^{8}	3.6×10^{7}
**8**	4.3×10^{7}	4.3×10^{7}	3.4×10^{7}
**10**	3.6×10^{7}	2.1×10^{7}	1.5×10^{7}

Table S13 (a). Concentration of free amino acids in Stevia fermentation broth at the beginning and end of fermentation

Free amino acid fermentation	Day 0-1	Fermentation broth of each strain with stevia on day 10 of fermentation (mg-L-1)（mg·L-1）						
		S1	S2	S3				
Total content	983.53 ±54.40	893.92 ±69.17	977.96 ±7.57	812.98 ±20.41				
Essential-amino acids：								
Therionine	17.72 ±1.77	37.21 ±10.29	35.62 ±5.84	63.76 ±6.24				
Valine	13.79 ±8.30	2.29 ±0.84	26.86 ±0.81	1.63 ±0.12				
Isoleucine	14.24 ±4.22	3.73 ±1.99	28.56 ±2.14	5.46 ±5.07				
Leucine	17.51 ±7.43	5.30 ±3.74	60.68 ±1.84	4.02 ±0.55				
Phenylalanine	31.34 ±21.95	9.13 ±2.83	98.30 ±22.03	12.13 ±4.02				

Lysine	24.07 ±11.26	10.28 ±0.84	18.06 ±2.96	2.38 ±0.36				
Methionine	18.36 ±7.58	11.34 ±2.74	25.33 ±2.74	12.19 ±2.17				

Note: Data are expressed as the mean ± standard deviation of three measurements.

Table S13 (b) Fermentation broth of each strain with steviosides

Free amino acid fermentation	Day 0-1	Fermentation broth of each strain with steviosides on day 10 of fermentation (mg-L-1)（mg·L-1）						
					S1	S2	S3	
Total content	983.53 ±54.40				784.89 ±32.08	795.68 ±5.44	800.36 ±24.31	
Essentia-lamino acids：								
Therionine	17.72 ±1.77				49.67 ±26.99	84.54 ±15.41	65.64 ±9.59	
Valine	13.79 ±8.30				23.01 ±3.35	23.64 ±4.03	5.51 ±2.65	
Isoleucine	14.24 ±4.22				44.91 ±3.70	30.49 ±3.21	12.33 ±4.70	
Leucine	17.51 ±7.43				13.26 ±5.25	24.00 ±5.97	22.11 ±7.91	
Phenylalanine	31.34 ±21.95				17.03 ±3.47	12.93 ±3.72	6.46 ±4.24	
Lysine	24.07 ±11.26				23.92 ±6.78	6.88 ±5.19	7.32 ±3.49	
Methionine	18.36 ±7.58				14.97 ±1.40	12.79 ±5.12	10.17 ±1.82	

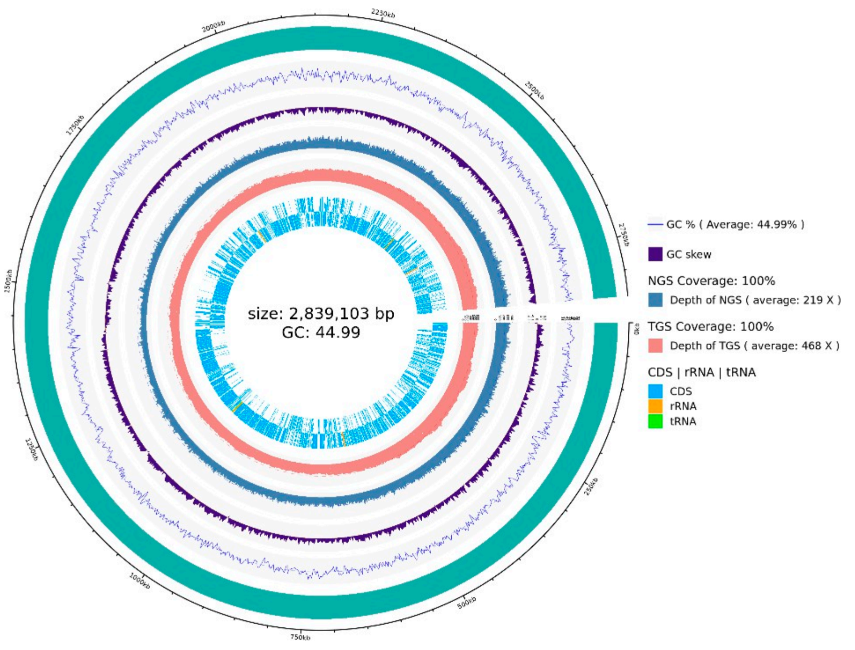
size: 2,839,103 bp
GC: 44.99
GC % (Average: 44.99%)
GC skew
NGS Coverage: 100%
Depth of NGS (average: 219 X)
TGS Coverage: 100%
Depth of TGS (average: 468 X)
CDS | rRNA | tRNA
CDS
rRNA
tRNA

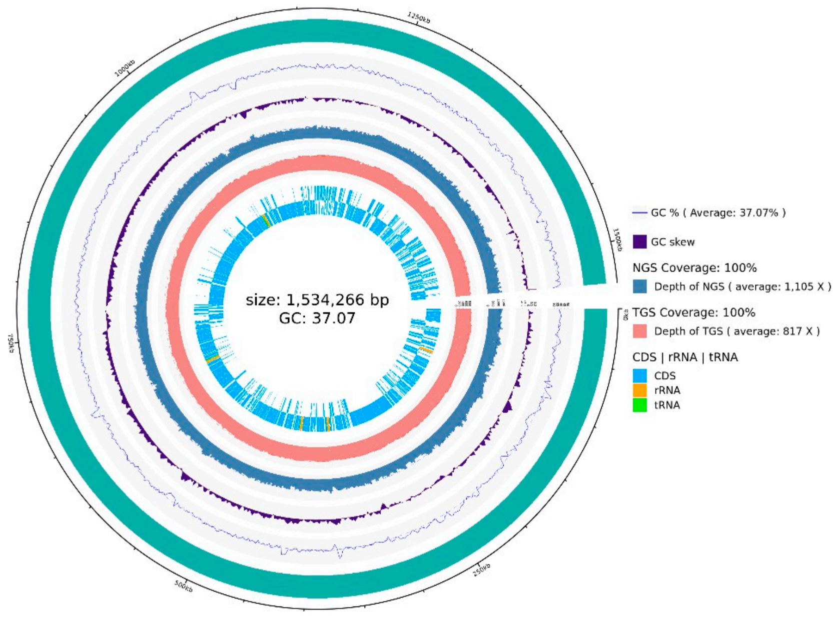
size: 1,534,266 bp
GC: 37.07
GC % (Average: 37.07%)
GC skew
NGS Coverage: 100%
Depth of NGS (average: 1,105 X)
TGS Coverage: 100%
Depth of TGS (average: 817 X)
CDS | rRNA | tRNA
CDS
rRNA
tRNA

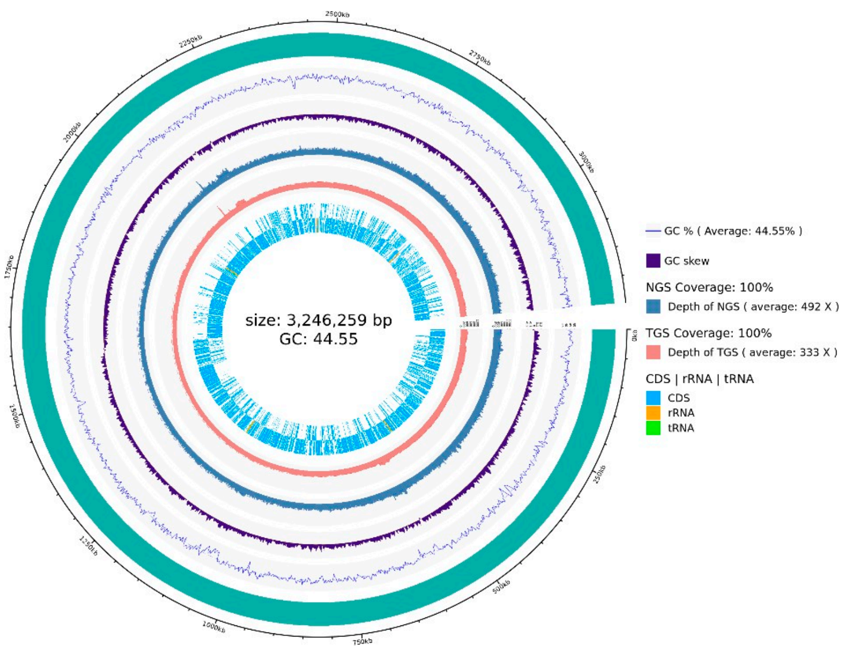

Figure S1. The complete genomes of three strains, S1, S2, and S3, consist of circular chromosomes with the following sizes: 1,534,266 bp, 2,839,103 bp, and 3,246,259 bp respectively.

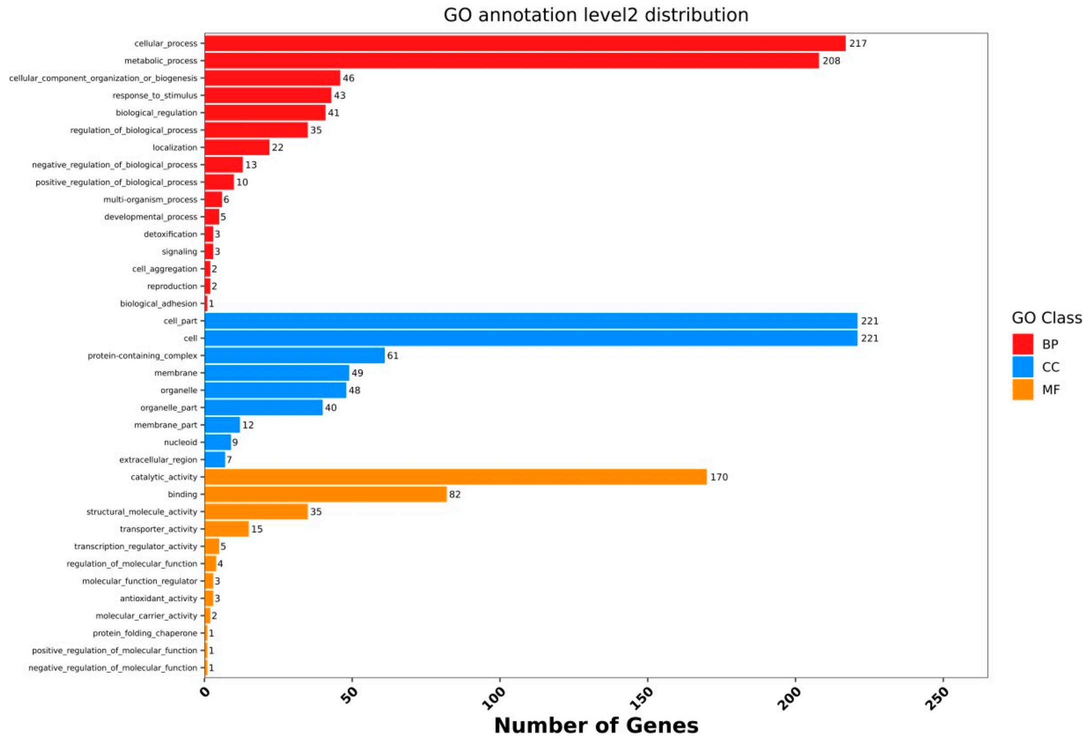

s

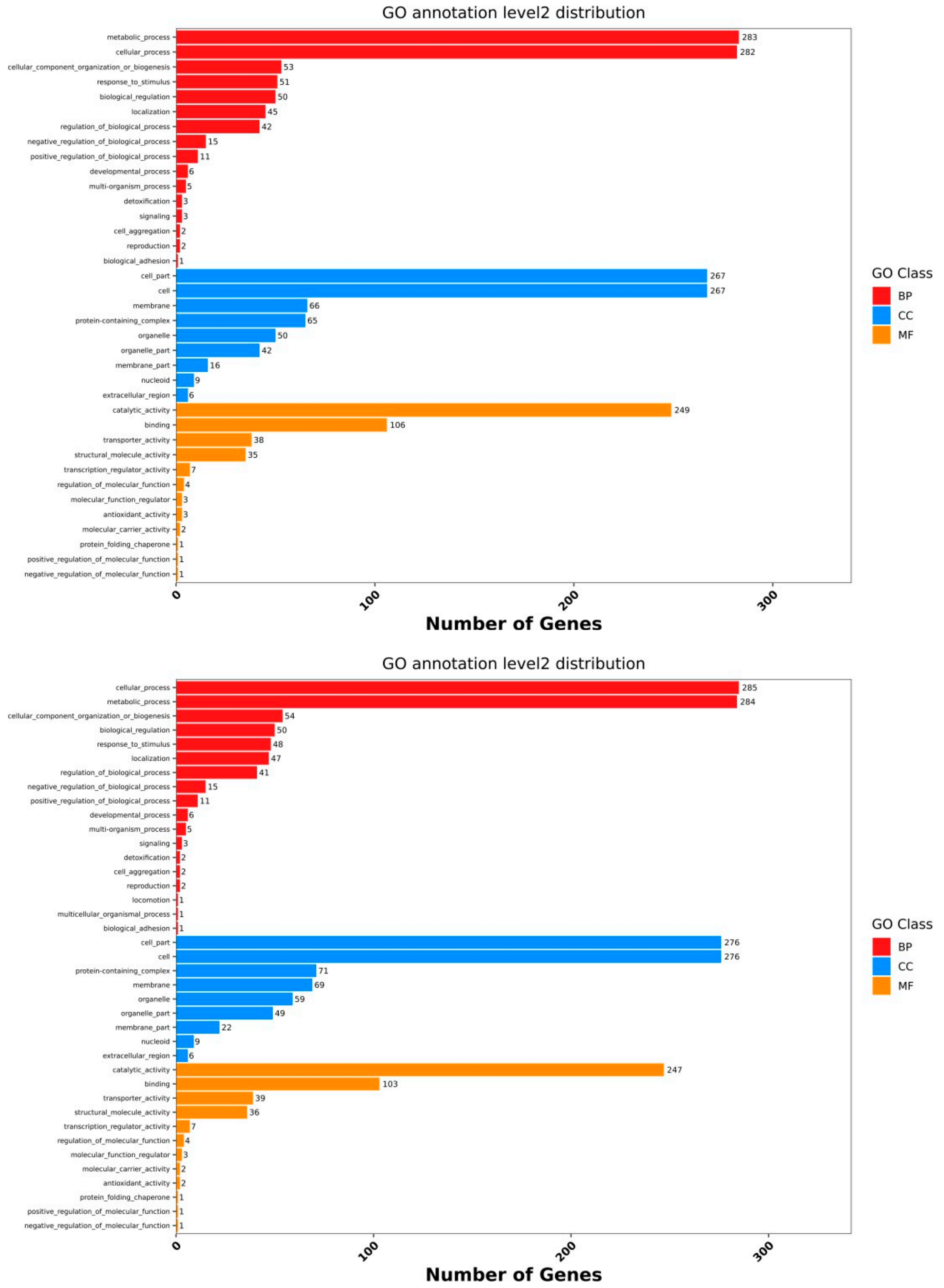

Figure S3. The GO classification system of *Apl. kunkee*i, *Lpb. Plantarum*, and *Lcb. pentosus* respectively.

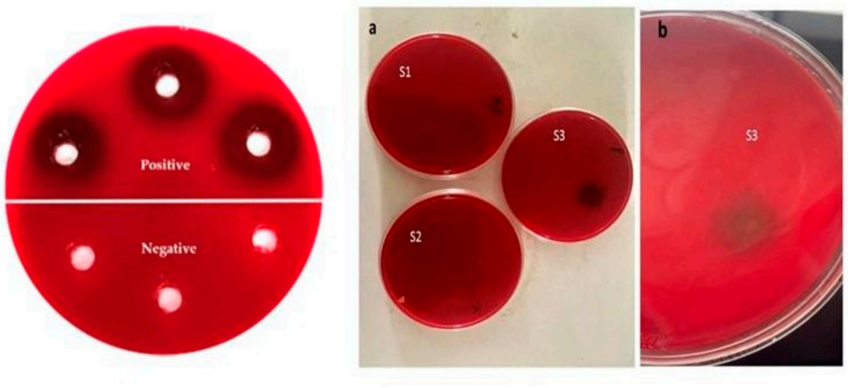

(**a**) Controls

Figure S4. (a) Hemolytic activity of three strains. (a) Isolates with a green halo were designated as α-hemolytic, (b) isolates presenting no halo were classified as γ-hemolytic. In our study demonstrates *Apl. kunkee*i and *Lpb. plantarum* shows γ –hemolysis. While *Lcb. pentosus S3* show α-hemolytic.

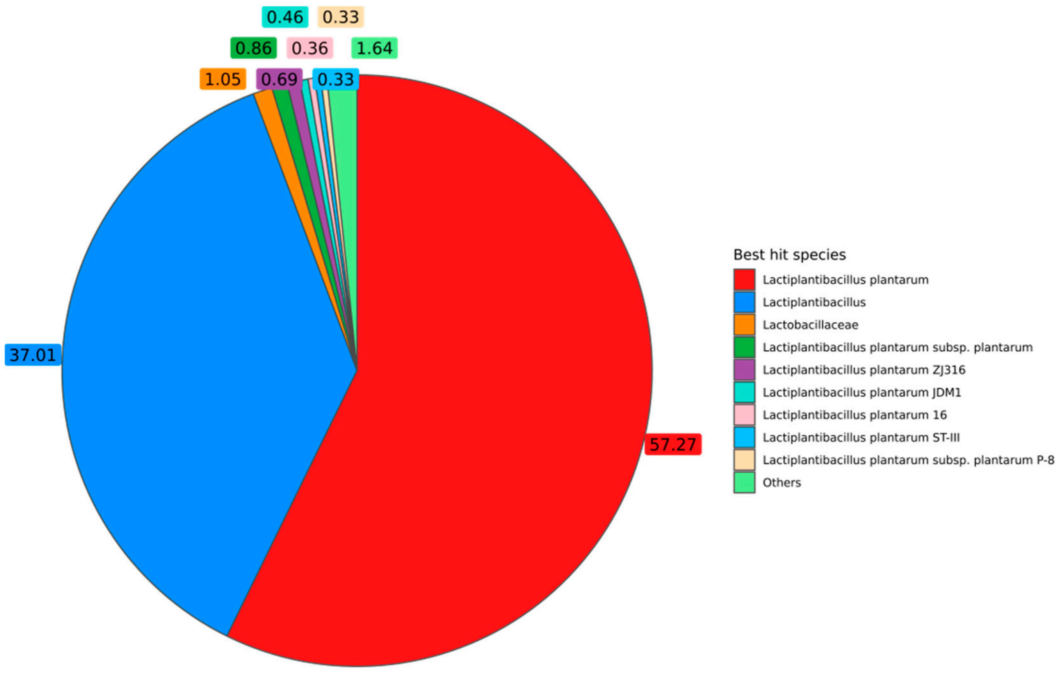

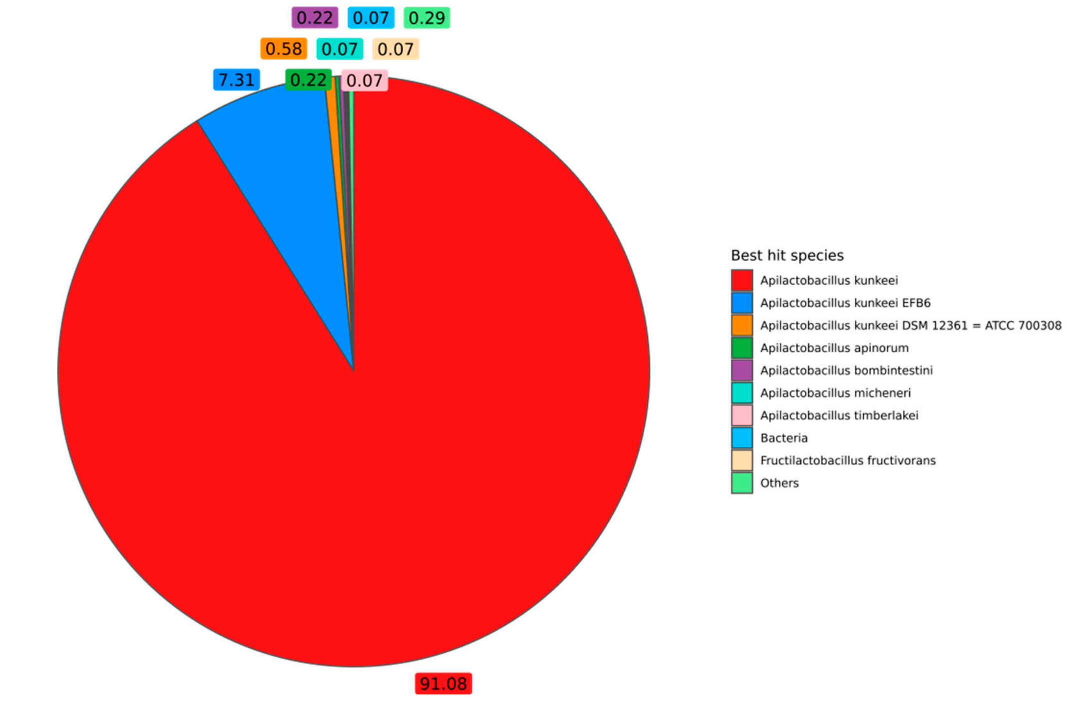

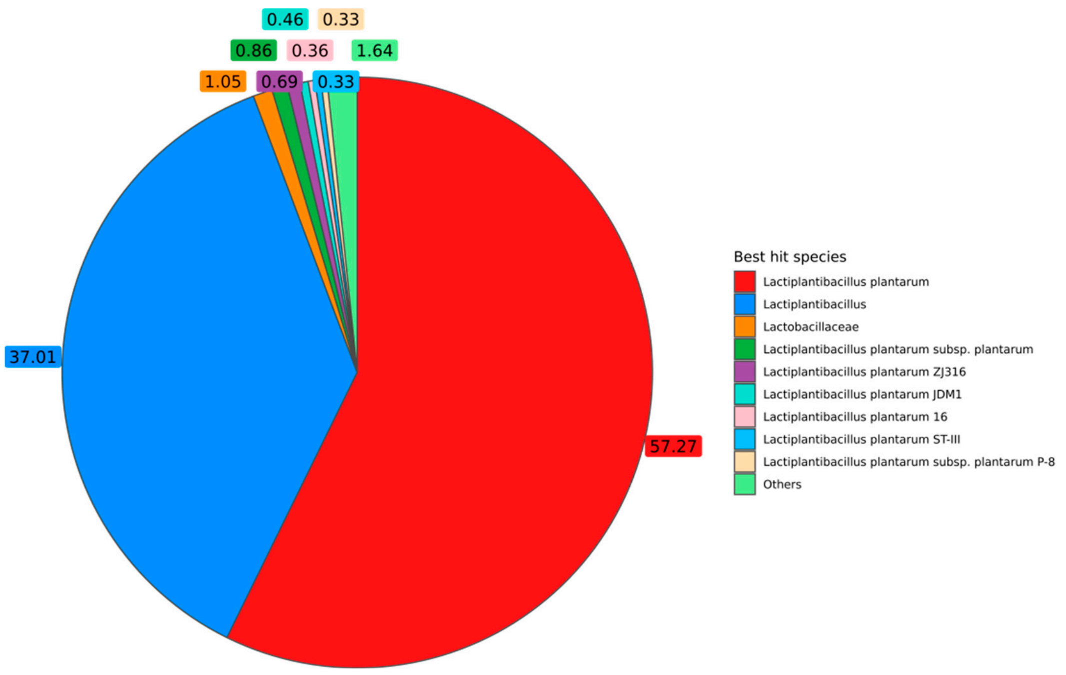

Figure S2 (a, b, c). Predicting the distribution of matching species in the database
